# Amphetamine Derivatives as Potent Central Nervous System Multitarget SERT/NET/H_3_ Agents: Synthesis and Biological Evaluation

**DOI:** 10.3390/molecules29225240

**Published:** 2024-11-06

**Authors:** Quxiang Li, Lili Ren, Dongli Wang, Junyong Luo, Changda Xu, Jian Feng, Yufan Qiu, Xiangqing Xu, Guoguang Chen

**Affiliations:** 1 School of Pharmacy, Nanjing Tech University, 30th South Puzhu Road, Nanjing 211816, China; liquxiang@163.com (Q.L.); renlili@njtech.edu.cn (L.R.); junyongluo@163.com (J.L.); 2 Jiangsu Key Laboratory of Central Nervous System Drug Research and Development, Institute of Pharmaceutical Research, Jiangsu Nhwa Pharmaceutical Co., Ltd., Xuzhou 221116, China; wangdongli@nhwa-group.com (D.W.); xuchangda@nhwa-group.com (C.X.); fengjian@nhwa-group.com (J.F.); 3 Pharmaron Beijing Co., Ltd., 6 Taihe Road, BDA, Beijing 100176, China; yufan.qiu@pharmaron-bj.com

**Keywords:** amphetamine, multitarget, antidepressant, molecular docking

## Abstract

In this research, a variety of novel amphetamine derivatives were synthesized and assessed for their potential as multifaceted antidepressant agents. Among these compounds, compound **11b** demonstrated potent inhibitory effects on both serotonin and noradrenaline transporters (SERT/NET) and high affinity for histamine H_3_ receptor (H_3_R), and displayed low affinity for off-target receptors (H1, α1) and hERG channels, which can reduce the prolongation of the QT interval. Molecular docking studies offered a rational binding model of compound **11b** when it forms a complex with SERT, NET, and the histamine H_3_ receptor. In vivo behavioral studies, compound **11b** dose-dependently reduced the immobility duration in the mouse FST and TST assays without a stimulatory effect on the locomotor activity. Furthermore, compound **11b** had a favorable pharmacokinetic profile in rats. Thus, compound **11b** has the potential to develop a novel class of drugs for the treatment of depression.

## 1. Introduction

Depression, impacting over 4.4% of individuals worldwide, is a prevalent and severe mental health condition marked by persistent feelings of sadness and a diminished interest in activities [1]. The World Health Organization anticipates that by 2030, depression will become the foremost contributor to disability [2]. A commonly accepted theory based on neurochemistry regarding depression is that it results from a deficiency or an imbalance of the biogenic amines noradrenaline (NE) and serotonin (5-HT) in the brain [3]. Therefore, current treatment methods for depression aim to boost the levels of neurotransmitters in the synaptic gap [4]. Medications known as selective serotonin reuptake inhibitors (SSRIs), including fluoxetine, sertraline, paroxetine, fluvoxamine, and citalopram, have become the primary treatment for depression. These drugs work by elevating the levels of serotonin in the synaptic cleft, thereby enhancing its availability for neurotransmission [5]. The serotonin and norepinephrine reuptake inhibitors (SNRIs), which are able to increase the levels of 5-HT and NE at the same time, have exhibited better therapeutic efficacy and higher rate of response when compared with single-target antidepressant (AD) agents [2]. SNRIs such as duloxetine, venlafaxine, and milnacipran have been established as first-line ADs in clinical settings, constituting the go-to therapy for depression [6]. Although SSRIs and SNRIs represented a major breakthrough in the treatment of depression, with enhanced safety and tolerability, there are still some troublesome issues. For example, the onset of their antidepressant activity is slow, and they can still cause certain undesirable side effects such as sexual dysfunction, nausea, headaches, and sleep disturbances [7,8]. Therefore, there remain large unmet medical requirements for the development of new ADs that offer the quicker onset of action and reduced incidence of side effects.

In recent years, a new trend has emerged in the development of ADs that can modulate multiple molecular targets, thereby generating beneficial effects through potential synergies [9,10]. Abundant evidence suggests that multitarget drugs, which selectively target monoamine transporters and other receptors, may possess a greater advantage in terms of efficacy, safety, and tolerability when compared to SSRIs or SNRIs [11,12]. The histamine H_3_ receptor (H_3_R), a G-protein coupled receptor, is abundantly expressed in crucial areas of the brain, such as the cerebral cortex, striatum, and hippocampus [13]. H_3_R functions as both an autoreceptor and a heteroreceptor and can modulate the levels of histamine and other neurotransmitters, thereby influencing the balance of different neurotransmitters [14,15]. Research has shown that H_3_R can interact with other depression-related transmitters (e.g., serotonin, noradrenaline, dopamine, and glutamate) to participate in the treatment or alleviation of depression through other neural circuits [16,17].

**Figure 1:**
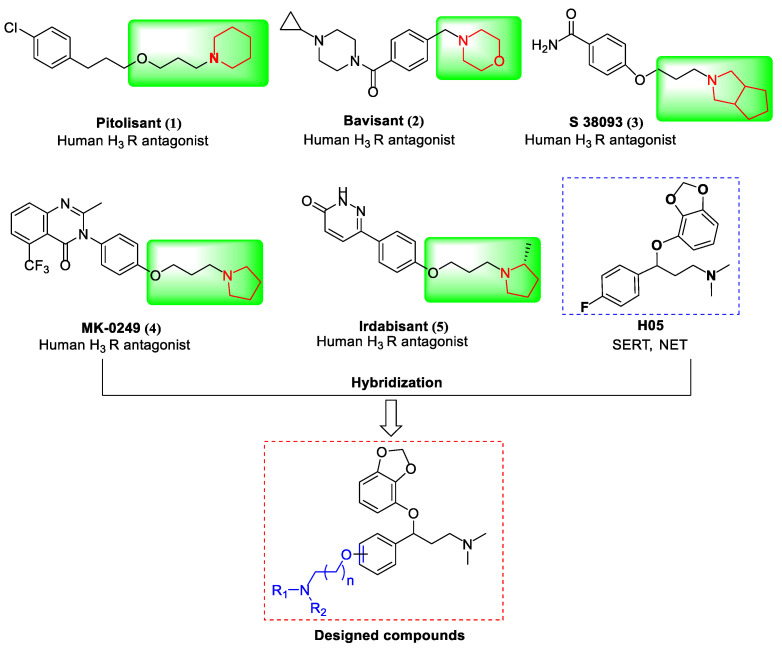
Representative H_3_R antagonists and design of new amphetamine derivatives.

Some preclinical compounds such as the H_3_R antagonist and 5-HT reuptake inhibitor have been developed to treat depression or improve cognitive impairment [4,18,19,20]. In our previous study, we described the biological evaluation of compound **H05** with very good inhibition activity for SERT and NET [21]. Animal behavioral studies indicated that compound **H05** displayed excellent antidepressant effects in the forced swim test and tail suspension test. Therefore, it was selected as a starting point for the design of further derivatives. In this paper, a series of amphetamine derivatives were designed by the approach of polypharmacological strategy and molecular hybridization method. The concept for designing new compounds is shown in Figure 1; compound **H05** was adopted as the primary scaffold, where the privileged fragments of reported H_3_R antagonists [22,23,24,25,26] (Pitolisant (**1**), Bavisant (**2**), S 38093 (**3**), MK-0249 (**4**), Irdabisant (**5**) (Figure 1)) were incorporated to construct the structure of new compounds for the purpose of modulating multiple receptors at the same time. Thus, it is hoped that the synergistic effect of H_3_ antagonists with serotonin and norepinephrine reuptake inhibitors could potentially provide more rapid therapeutic advantages for suffering from depression.

A series of new compounds in Table 1 was synthesized, and we evaluated their pharmacological efficacy and relative affinity for the multiple receptors, respectively. Among these compounds, the structure–activity relationship (SAR) studies showed that compound **11b** presented a favorable polypharmacological antidepressive profile.

In vitro, compound **11b** displayed much higher potency for the desired targets (SERT, NET, and H_3_) compared with other off-target receptors (H_1_, α_1_, and hERG channels). Further in vivo behavioral studies indicated that it has favorable effects in FST and TST without a stimulating influence on the locomotor activity. Moreover, compound **11b** exhibited favorable pharmacokinetic properties. Thus, compound **11b** has the potential to be developed as a novel antidepressant candidate to treat depression.

## 2. Results and Discussion

### 2.1. Chemistry

**Scheme 1:**
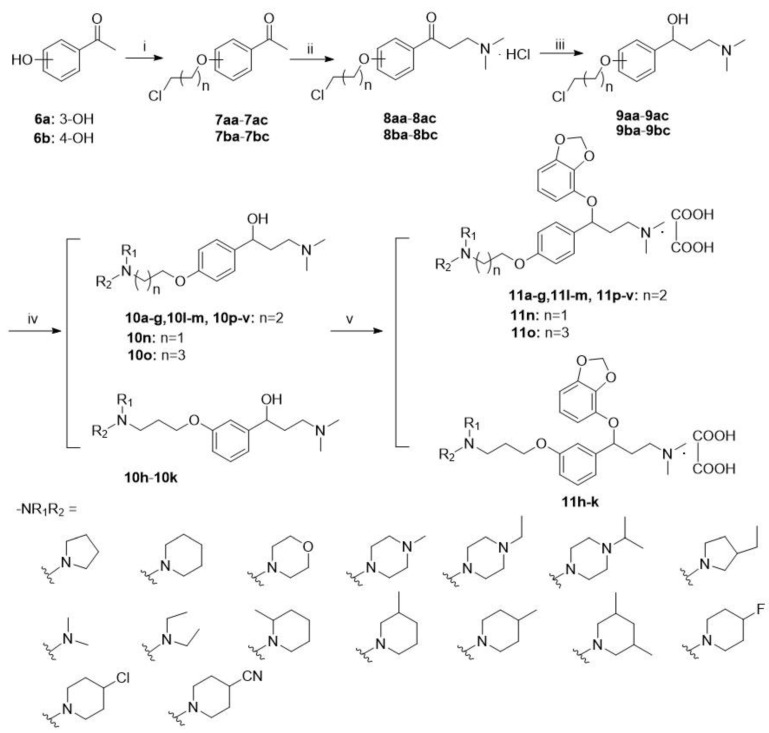
Reagents and conditions: (i) Appropriate bromochloro alkanes, K_2_CO_3_, acetone, reflux, 10 h; (ii) polyformaldehyde, dimethylamine hydrochloride, EtOH, reflux, 15 h; (iii) NaBH_4_, NaOH/H_2_O, MeOH, 0 °C, 3 h; (iv) secondary amines, K_2_CO_3_, KI, DMF, 85 °C, 8 h; (v) (a) 4-fluorobenzo[d][1,3]dioxole, t-BuOK, DMSO, 60 °C, 5 h; (b) oxalic acid, EA, rt.

The synthesis of the new amphetamine derivatives is summarized in Scheme 1. As shown in Scheme 1, compounds **7aa**–**7ac** and **7ba**–**7bc** were obtained through alkylation of **6a**–**6b** with 1-bromo-2-chloroethane, 1-bromo-3-chloropropane, and 1-bromo-4-chlorobutane. Mannich reaction of **7aa**–**7ac** and **7ba**–**7bc** with paraformaldehyde and dimethylamine hydrochloride gave intermediates **8aa**–**8ac** and **8ba**–**8bc**, followed by reduction with sodium borohydride to afford the related alcohol **9aa**–**9ac** and **9ba**–**9bc**. Compounds **10a**–**10v** were prepared by coupling **9aa**–**9ac** and **9ba**–**9bc** with different secondary amines in the presence of K_2_CO_3_ in DMF. Subsequently, the intermediates **10a**–**10v** were coupled with 4-fluorobenzo[d][1,3]dioxolane to afford compounds **11a**–**11v** as free base, and further salification yielded target compounds such as oxalic acid salt.

### 2.2. In Vitro Study of Target Compounds

In this work, our initial focus was to investigate the effect of different amine moieties for SERT, NET, and H_3_ receptors (Table 1, compounds **11a**–**v**). As shown in Table 1, compounds **11a** (pyrrolidine) and **11b** (piperidine) showed high affinity for SERT, NET, and H_3_ receptors (SERT, *K*_i_ = 10.3 nM; NET, *K*_i_ = 19.2 nM; H_3_, *K*_i_ = 4.2 nM and SERT, *K*_i_ = 7.8 nM; NET, *K*_i_ = 10.5 nM; H_3_, *K*_i_ = 5.5 nM, respectively). Compound **11c**, bearing morpholinyl substitution, displayed moderate affinity for three receptors. The compounds with *N*-methyl piperazinyl, *N*-ethyl piperazinyl, and *N*-isopropyl piperazinyl substitution (**11d**–**11f**) exhibited moderate inhibition activity against SERT and NET, but showed weak affinity for H_3_ receptor. Compound **11g**, bearing cyclopenta[c]pyrrole moiety, displayed moderate affinity for SERT and NET (SERT, *K*_i_ = 31.3 nM; NET, *K*_i_ = 48.2 nM) and high affinity for H_3_ receptor (H_3_, *K*_i_ = 10.6 nM). When the propyl amine moieties were introduced at the 2-position on the benzene ring (Table 1), compounds **11h**–**11k** decreased the affinity for all the three receptors compared with **11a**–**11c** and **11f**. The amine moieties dimethylamine and diethylamine (compounds **11l**–**11m**) showed high affinity for SERT (*K*_i_ = 17.1 nM and *K*_i_ = 28.4 nM) and moderate activity for NET (*K*_i_ = 114.9 nM and *K*_i_ = 85.2 nM), but almost lost the affinity for the H_3_ receptor.

According to the above results, compound **11b**, bearing a three-carbon chain, exhibited preferable affinity for all three receptors. Therefore, we aimed to ascertain the impact of the length of the linker connecting the phenyl group and the piperidine ring. As shown in Table 1, chain lengths of two carbon atoms (**11n**, *K*i = 1021.5 nM) or four (**11o**, 835.3 nM) resulted in significantly reduced H_3_ receptor binding, but presented moderate affinity for SERT and NET. Compounds **11p**–**11s** remained high affinity for three targets when the piperidine ring was substituted with electron-donating groups (such as -CH_3_ or -di-CH_3_) compared with compound **11b**. Especially, compound **11q**, bearing the 2-methylpiperidyl moiety, displayed higher affinity for the three receptors (SERT, *K*_i_ = 15.1 nM; NET, *K*_i_ = 20.7 nM; H_3_, *K*_i_ = 3.2 nM) than compounds **11p**, **11r**, and **11s**. When the piperidine ring was substituted with electron-withdrawing groups, such as 4-fluopiperidine (**11t**), 4-chloropiperidine (**11u**), and 4-cyanopiperidine (**11v**), all of the three compounds failed to improve the affinities for the SERT, NET, and H_3_ receptors compared to compound **11b**.

Overall, compounds **11a**, **11b**, and **11q** exhibited high binding affinity for SERT, NET, and H_3_ receptors (SERT, *K*_i_ < 15 nM; NET, *K*_i_ < 25 nM; H_3_, *K*_i_ < 10 nM), and the potency ratio among SERT, NET, and H_3_ was less than 10-fold, indicating that these compounds possessed balanced receptor activity profiles. Thus, compounds **11a**, **11b**, and **11q** were chosen for further investigation to measure their affinity for the H_1_ and α_1_ receptors.

Some studies have demonstrated that several ADs can generate adverse effects when used for treating depressive disorders, like weight gain, sedation, blood pressure problems, and QTc prolongation [27,28,29,30]. Evidence has shown that these side effects are linked to the activities of several off-target receptors, namely, histamine H_1_, adrenergic α_1_, and hERG. For instance, the antagonism of the H_1_ receptor may result in weight gain and sedation, while the blockade of the α_1_ adrenergic receptor gives rise to orthostatic hypotension [31]. Hence, the three aforementioned compounds were subjected to further assessment regarding these receptors in the present study. As depicted in Table 2, compounds **11a**, **11b**, and **11q** displayed affinities that varied from low to moderate for the H_1_ and α_1_ receptors. This indicates that these compounds are less prone to trigger adverse effects related to the treatment.

Cardiotoxicity, which is a significant adverse effect, is often caused by the blockade of the hERG potassium channel that controls the normal heart rhythm [32]. Consequently, hERG inhibition has become a widely recognized biomarker for the evaluation of the cardiotoxicity of candidate drugs. As illustrated in Table 2, compound **11b** (with an IC_50_ value of 1000.8 nM) displayed lower hERG-inhibiting activity compared to the rest of compounds, suggesting that compound **11b** had a low risk of QT interval prolongation.

Based on the above results, compound **11b** showed higher affinity for SERT, NET, and H_3_ receptors and lower affinity for H_1_ and α_1_ receptors and hERG than other compounds. Therefore, compound **11b** was worthy of further investigation.

### 2.3. Molecular Docking

**Figure 2:**
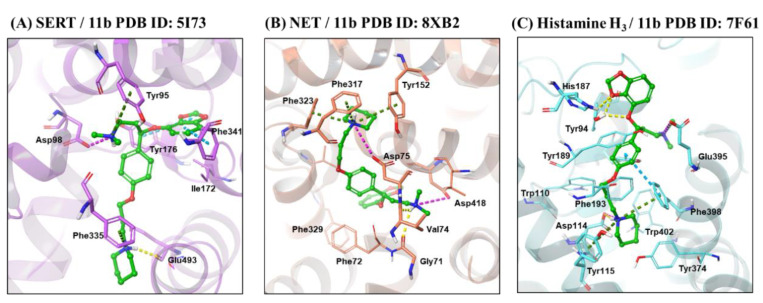
Binding modes of compound **11b** with active sites of SERT ((**A**) PDB code: 5I73), NET ((**B**) PDB code: 8XB2) and histamine H_3_ ((**C**) PDB code: 7F61). The ligands are shown as balls and sticks. Hydrogen bonds, salt bridges, pi–pi stacking, and cation–pi are shown as dotted lines with yellow, magenta, cyan, and green color.

The docking results of compound **11b** with targeted receptor proteins (5I73, 8XB2, and 7F61) are illustrated in Figure 2A–C. As shown in Figure 2A, for serotonin transporter (SERT), Asp98 and Glu493 established salt bridge interactions with the basic nitrogens while Tyr95 and Phe335 formed cation–pi interactions. Tyr176 and Phe341 further formed pi–pi interaction with the benzodioxole group. For NET-compound **11b** complex (Figure 2B), Asp75 and Asp418 formed salt bridge interactions with the two basic nitrogens of compound **11b**. Three aromatic residues, Tyr152, Phe317, and Phe323, formed cation–pi interactions with the piperidine nitrogen. Hydrogen bonds were also observed between the carbonyl groups of Gly71 and Val74 and the dimethylamino group of compound **11b**. For histamine H_3_ receptor (Figure 2C), Asp114 and Glu395 formed salt bridge interactions with the two basic nitrogens of compound **11b**. Additionally, three hydrogen bond interactions were observed between the benzodioxole group and the sidechains of Tyr94 and His187. In addition, the nitrogen of piperidine participated in the formation of a cation–pi interaction with aromatic residues Tyr115 and Phe398, while a pi–pi interaction was observed between Phe398 and the phenyl ring of compound **11b**. Other aromatic residues, including Trp110, Phe193, Tyr189, Tyr374, and Trp403, formed hydrophobic interactions with compound **11b**. Compared with SERT and NET, it seems that more hydrophobic interactions caused by clusters of pocket aromatic residues may contribute to higher activity over the histamine H_3_ receptor.

### 2.4. Intrinsic Activity of Compound **11b**

Compound **11b** was selected for further analysis because of its exceptional in vitro results and favorable safety profile. As shown in Table 3, compound **11b** exhibited weak agonist activity on the H_3_ receptor, with an efficacy less than 10% of that of the reference compound. In the antagonist assay, compound **11b** blocked more than 90% of the activity of the three targets. Therefore, compound **11b** acted as an antagonist of SERT (IC_50_ = 20.1 nM), NET (IC_50_ = 49.6 nM), and H_3_ (IC_50_ = 0.72 nM).

### 2.5. Acute Toxicity

The acute toxicity in vivo was evaluated by measuring LD_50_ values. Compound **11b** (with an LD_50_ value of 512 mg/kg in females) manifested a higher safety threshold when compared to duloxetine (LD_50_ = 279 mg/kg [33], females), which indicates that compound **11b** has an excellent safety profile and low acute toxicity.

### 2.6. In Vivo Behavioral Studies

Based on the above in vitro and acute toxicity results, compound **11b** was initially screened for in vivo activity by utilizing models of TST and FST. These models are the classical behavioral screening tools for detecting potential antidepressant drugs [34,35]. Additionally, a locomotor activity test was also carried out to identify the false positive effects in the animal models of depression.

#### 2.6.1. Forced Swimming Test (FST) and Tail Suspension Test (TST) in Mice

**Figure 3:**
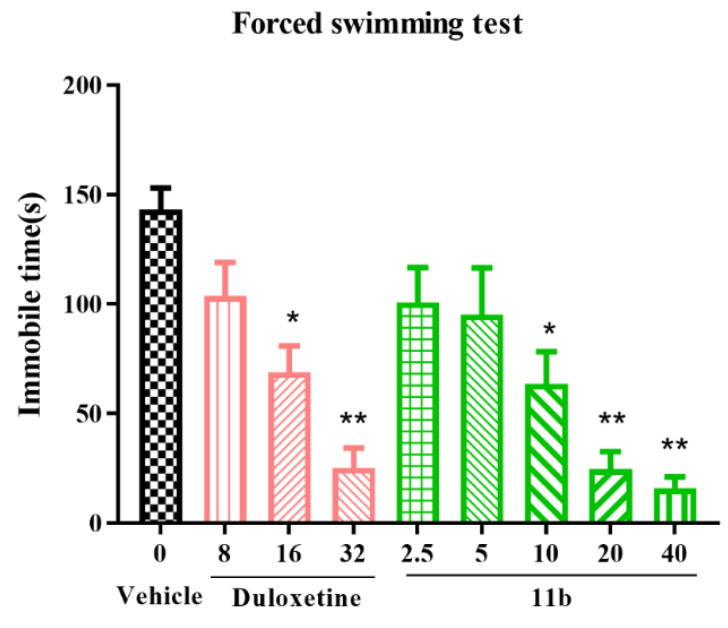
Antidepressant-like effects of compound **11b** in the forced swimming test (FST) in mice. Results are represented as mean ± SEM. One-way ANOVA followed by Dunnett’s test: * *p* < 0.05, ** *p* < 0.01, compared with the vehicle control group (*n* = 16).

**Figure 4:**
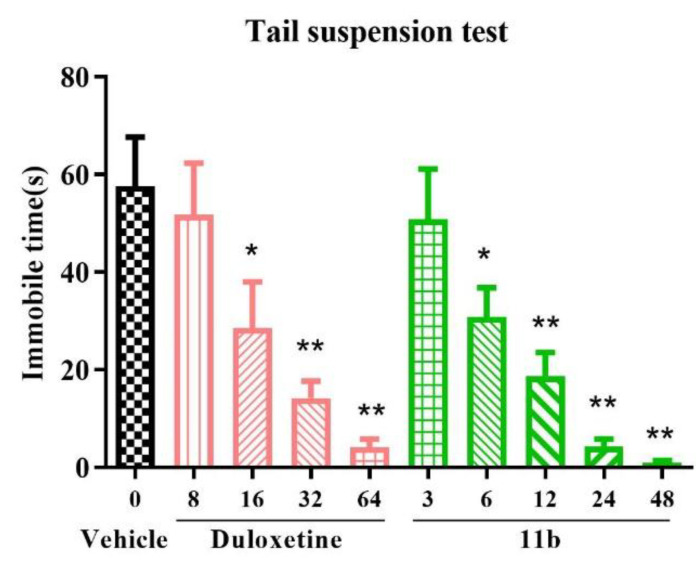
Antidepressant-like effects of compound **11b** in the tail suspension test (TST) in mice. Results are represented as mean ± SEM. One-way ANOVA followed by Dunnett’s test: * *p* < 0.05, ** *p* < 0.01, compared with the vehicle control group (*n* = 16).

As shown in Figure 3 and Figure 4, oral administration of duloxetine at doses of 8, 16, and 32 mg/kg brought about a dose-dependent reduction in the duration of immobility with minimal effective doses (MEDs) of 16 mg/kg in TST and 16 mg/kg in FST, respectively.

Compound **11b** induced a remarkable decrease in immobility time in a dose-dependent manner. In contrast to the control group, it exhibited a lower MED, namely, 10 mg/kg in TST and 6 mg/kg in FST, which indicated that compound **11b** was more effective than duloxetine in these models.

#### 2.6.2. Locomotor Activity Test in Mice

**Figure 5:**
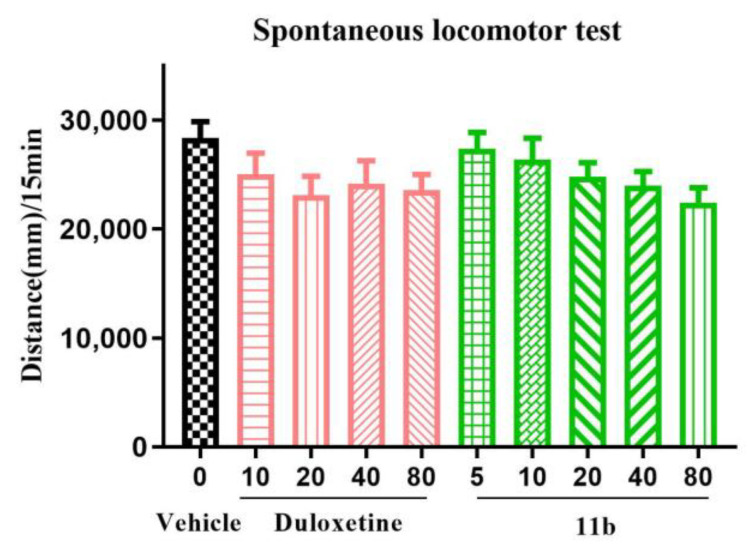
Effects of compound **11b** and duloxetine on the spontaneous locomotor activity in mice. Results are represented as mean ± SEM. One-way ANOVA followed by Dunnett’s test: compared with the vehicle control group (*n* = 16).

In order to determine if drug-induced changes in locomotor activity contributed to the behaviors observed in TST and FST, the locomotor activity test of compound **11b** was carried out. As depicted in Figure 5, compound **11b** did not significantly alter the total travel distance at doses of 5, 10, 20, 40, and 80 mg/kg within the 15 min period, which likely rules out false positive antidepressant effects.

### 2.7. Selectivity Characterization of Compound **11b**

We evaluated the interactions of compound **11b** with other receptors associated with central nervous system (CNS) disorders. A selectivity profile was then constructed by employing additional receptors such as DAT, D_1_, D_2_, D_3_, 5HT_2A_, 5HT_2C_, α_2_, H_2_, H_4_, M_1_–M_5_, σ_1_, σ_2_, and NMDA receptors. Compound **11b** demonstrated a moderate affinity for 5HT_2A_ receptor (*K*_i_ = 320 nM), while it exhibited no significant affinity (*K*_i_ > 1000 nM) for the other potential targets (see Supplementary Materials).

### 2.8. Pharmacokinetic Study in Rats

The pharmacokinetic properties of compound **11b** were explored in rats (Table 4). When compound **11b** was intravenously administered to rats at a dose of 2 mg/kg (*n* = 6), detectable plasma levels were obtained, with a half-life (t_1/2_) of 1.3 h. On the other hand, oral administration of compound **11b** to rats at a dose of 20 mg/kg (*n* = 6) led to a t_1/2_ of 2.6 h. Following intravenous administration of compound **11b**, the area under the curve (AUC) value was 265.1 ng·h/mL. After oral administration, the AUC value of compound **11b** was 944.1 ng·h/mL. The C_max_ value after oral dosing was 133.9 ng/mL, and the T_max_ value was 2.0 h. The bioavailability of compound **11b** was 35.6%. In sum, compound **11b** exhibited a desirable drug-like pharmacokinetic profile.

## 3. Materials and Methods

### 3.1. General Information

All commercially available chemicals and reagents were used without further purification. All reagents were of analytical grade or of chemical purity (>95%). Melting points were determined in open capillary tubes and were uncorrected. ^1^H NMR spectra were recorded on a Bruker Advance III 400 spectrometer (Bruker, Karlsruhe, Germany) at 400 MHz (^1^H) using DMSO-*d*_6_ as solvent. Chemical shifts are given in *δ* values (ppm), using tetramethylsilane (TMS) as the internal standard; coupling constants (*J*) are given in Hz. Signal multiplicities were characterized as s (singlet), d (doublet), t (triplet), q (quartet), m (multiplex), br (broad signal). Analytical thin layer chromatography (TLC) was performed on silica gel GF254. Column chromatographic purification was carried out using silica gel. Compound purity was determined by high-performance liquid chromatography (HPLC), and all final test compounds displayed purity higher than 95%.

### 3.2. Instrumentation

HPLC methods used the following: Shimadzu LC-20AD spectrometer (Shimadzu, Kyoto, Japan); column, Agilent Eclipse Plus C18 (4.6 mm × 250 mm, 5 μm, Agilent, Santa Clara, USA); mobile phase A: 0.02 mol/L NH_4_H_2_PO_4_ (0.2%Et_3_N, pH = 3) aq./acetonitrile = 90/10; mobile phase B: 0.02 mol/L NH_4_H_2_PO_4_ (0.2%Et_3_N, pH = 3) aq./acetonitrile = 10/90; flow rate, 1 mL/min; sample size, 10 µL; column temperature, 35 °C. UV detection was performed at 210 nm. HRMS methods: Agilent 6530 Q-TOF LC/MS, column X BridgeR Shield RP18 (4.6 × 150 mm, 3.5 µm, Waters, Milford, MA, USA), C18; mobile phase: A, 5 mmol/L NH4OAc (pH = 6.5) (20:80); B, MeOH; eluent, 20% A and 80% B (*V*:*V*); flow rate, 1.0 mL/min; column temperature, 40 °C; UV detection, 210 nm.

### 3.3. Synthesis

#### 3.3.1. General Procedure for the Preparation of Intermediates **7**

The mixture of compound **6a** or **6b** (0.1 mol), K_2_CO_3_ (0.2 mol), acetone (150 mL), and corresponding bromochloro alkanes (0.12 mol) was heated to 60 °C for 10 h. After filtering, the filtrate was concentrated under reduced pressure. The residue was extracted with 75 mL of dichloromethane three times after it was diluted with 100 mL of water. After drying over MgSO_4_, dichloromethane was removed under reduced pressure and the crude product was separated by silica gel column chromatography to afford **7aa**–**7ac** and **7ba**–**7bc**.

#### 3.3.2. General Procedure for the Preparation of Intermediates **8**

The **7aa**–**7ac** or **7ba**–**7bc** (50 mmol), para-formaldehyde (75 mmol), and dimethyl amine hydrochloride (75 mmol) were suspended in 100 mL of ethanol. Then, 0.2 mL of concentrated hydrochloric acid was added, and the resulting mixture was heated until it refluxed for 15 h. Next, the mixture was cooled to room temperature. The precipitate that formed was collected through filtration and then washed with 100 mL of anhydrous ethanol, thus affording the intermediates **8aa**–**8ac** or **8ba**–**8bc**.

#### 3.3.3. General Procedure for the Preparation of Intermediates **9**

Sodium borohydride (15 mmol) was slowly added to a methanol (100 mL) solution containing **8aa**–**8ac** or **8ba**–**8bc** (30 mmol) and 5% sodium hydroxide (15 mL) at 0 °C. Three hours later, the reaction mixture was evaporated under reduced pressure. Then, water was used to dilute the residue, and ethyl acetate was employed for extraction. The organic layer obtained was washed with brine, dried with MgSO_4_, and concentrated in vacuo to yield intermediate **9aa**–**9ac** or **9ba**–**9bc**.

#### 3.3.4. General Procedure for the Preparation of Intermediates **10**

To a suspension of intermediate **9aa**–**9ac** or **9ba**–**9bc** in DMF, 2 equivalents of K_2_CO_3_, a catalytic amount of KI, and corresponding secondary amines were added, and the mixture was stirred at 85 °C for 8 h and then filtered. The filtrate was diluted with water and extracted with dichloromethane. The organic phase was washed with water, and dried over Na_2_SO_4_, and then dichloromethane was evaporated in vacuo. The crude mixture was purified by silica gel column chromatography to afford **10a**–**10v**.

#### 3.3.5. General Procedure for the Preparation of Target Compound **11**

Potassium tert-butoxide (15 mmol) was added portion-wise to a suspension of intermediate **10a**–**10v** (10 mmol) and 4-fluorobenzo[d][1,3]dioxole (15 mmol) in DMSO (50 mL). The resultant mixture was heated to 60 °C and maintained at this temperature for 5 h. Once cooled to room temperature, the reaction mixture was quenched with 75 mL of water and then extracted with ethyl acetate. The organic extract was washed with brine, dried over anhydrous MgSO_4_, and concentrated under reduced pressure. The crude product was further purified through column chromatography, affording a light-yellow oil. Subsequently, the free base was treated with an appropriate quantity of oxalic acid to obtain the target compounds **11a**–**11v**.

*3-(benzo[d][1,3]dioxol-4-yloxy)-N,N-dimethyl-3-(4-(3-(pyrrolidin-1-yl)propoxy)phenyl)propan-1-amine oxalate* (**11a**), Pale-white solid; m.p. 138–140 °C; yield 51.2%; ^1^H NMR (400 MHz, DMSO-*d*_6_) δ 7.33 (d, *J* = 8.2 Hz, 1H), 7.28 (d, *J* = 8.1 Hz, 1H), 6.92 (d, *J* = 8.0 Hz, 2H), 6.64 (t, *J* = 8.1 Hz, 1H), 6.54–6.38 (m, 2H), 6.01–5.89 (m, 2H), 5.45 (dd, *J* = 8.3, 4.6 Hz, 1H), 4.04 (p, *J* = 6.9, 6.0 Hz, 2H), 3.41–3.16 (m, 6H), 3.06 (tp, *J* = 13.3, 7.7, 6.3 Hz, 2H), 2.73 (d, *J* = 8.9 Hz, 6H), 2.47–2.26 (m, 1H), 2.24–2.02 (m, 3H), 1.99–1.85 (m, 4H). ^13^C NMR (101 MHz, DMSO-*d*_6_) δ 165.24, 158.50, 148.94, 141.69, 136.24, 132.72, 128.07, 122.24, 114.95, 111.75, 103.05, 101.34, 78.21, 65.29, 54.22, 53.42, 51.80, 42.73, 32.71, 25.77, 23.16. HRMS (ESI) *m*/*z*: calcd for C_25_H_34_N_2_O_4_ [M + H]^+^: 427.2591; found: 427.2592.
*3-(benzo[d][1,3]dioxol-4-yloxy)-N,N-dimethyl-3-(4-(3-(piperidin-1-yl)propoxy)phenyl)propan-1-amine oxalate* (**11b**), Pale-white solid; m.p. 105–107 °C; yield 43.5%; ^1^H NMR (400 MHz, DMSO-*d*_6_) δ 7.33 (d, *J* = 8.3 Hz, 2H), 6.91 (d, *J* = 8.2 Hz, 2H), 6.65 (t, *J* = 8.1 Hz, 1H), 6.50 (dd, *J* = 13.3, 8.1 Hz, 2H), 5.97 (d, *J* = 12.1 Hz, 2H), 5.42 (dd, *J* = 8.3, 4.6 Hz, 1H), 4.01 (q, *J* = 7.7, 6.1 Hz, 2H), 3.11 (qt, *J* = 18.2, 4.6 Hz, 8H), 2.72 (s, 6H), 2.40–2.26 (m, 1H), 2.11 (qd, *J* = 11.8, 5.7 Hz, 3H), 1.73 (p, *J* = 5.5 Hz, 4H), 1.61–1.47 (m, 2H). ^13^C NMR (100 MHz, DMSO-*d*_6_) δ 158.48, 148.93, 141.69, 136.22, 132.72, 128.07, 122.25, 114.94, 111.71, 103.04, 101.34, 78.20, 65.46, 54.19, 53.87, 52.50, 42.71, 32.70, 23.86, 22.99, 21.92. HRMS (ESI) *m*/*z*: calcd for: C_26_H_36_N_2_O_4_ [M + H]^+^: 441.2748; found:441.2739.
*3-(benzo[d][1,3]dioxol-4-yloxy)-N,N-dimethyl-3-(4-(3-morpholinopropoxy)phenyl)propan-1-amine oxalate* (**11c**), Pale-white solid; m.p. 169–170 °C; yield 35.4%; ^1^H NMR (400 MHz, DMSO-*d*_6_) δ 7.32 (d, *J* = 8.4 Hz, 2H), 6.91 (d, *J* = 8.4 Hz, 2H), 6.65 (t, *J* = 8.1 Hz, 1H), 6.50 (ddd, *J* = 13.5, 8.2, 1.0 Hz, 2H), 5.97 (dd, *J* = 12.1, 1.0 Hz, 2H), 5.42 (dd, *J* = 8.3, 4.6 Hz, 1H), 3.98 (t, *J* = 6.2 Hz, 2H), 3.23 (ddt, *J* = 28.3, 11.8, 5.7 Hz, 2H), 3.12–2.97 (m, 8H), 2.75 (s, 6H), 2.63 (t, *J* = 7.3 Hz, 2H), 2.34 (d, *J* = 10.1 Hz, 1H), 2.16 (d, *J* = 11.9 Hz, 1H), 1.91 (p, *J* = 6.7 Hz, 2H). ^13^C NMR (101 MHz, DMSO-*d*_6_) δ 158.59, 148.94, 141.67, 136.23, 132.60, 128.07, 122.25, 114.94, 111.73, 103.06, 101.35, 78.18, 65.58, 64.55, 54.21 (d, *J* = 10.6 Hz), 52.10, 42.64, 32.60, 24.26. HRMS (ESI) *m*/*z*: calcd for: C_25_H_34_N_2_O_5_ [M + H]^+^: 443.2540; found:443.2545.
*3-(benzo[d][1,3]dioxol-4-yloxy)-N,N-dimethyl-3-(4-(3-(4-methylpiperazin-1-yl)propoxy)phenyl)propan-1-amine oxalate* (**11d**), Pale-white solid; m.p. 155–156 °C; yield 40.8%; ^1^H NMR (400 MHz, DMSO-*d*_6_) δ 7.32 (d, *J* = 8.2 Hz, 2H), 6.90 (d, *J* = 8.2 Hz, 2H), 6.65 (t, *J* = 8.1 Hz, 1H), 6.50 (dd, *J* = 15.6, 8.1 Hz, 2H), 5.98 (d, *J* = 12.5 Hz, 2H), 5.42 (dd, *J* = 8.3, 4.6 Hz, 1H), 3.97 (t, *J* = 6.2 Hz, 2H), 3.19 (dt, *J* = 11.8, 6.1 Hz, 2H), 3.11–2.97 (m, 4H), 2.76 (s, 9H), 2.61 (d, *J* = 10.6 Hz, 6H), 2.33 (d, *J* = 10.8 Hz, 1H), 2.14 (t, *J* = 12.1 Hz, 1H), 1.90 (p, *J* = 6.5 Hz, 2H). ^13^C NMR (101 MHz, DMSO-*d*_6_) δ 158.76, 148.93, 141.67, 136.22, 132.43, 128.06, 122.24, 114.91, 111.72, 103.05, 101.34, 78.17, 65.83, 54.19, 53.84, 52.69, 50.12, 43.09, 42.65, 32.62, 25.95. HRMS (ESI) *m*/*z*: calcd for: C_26_H_37_N_3_O_4_ [M + H]^+^: 456.2857; found:456.2851.
*3-(benzo[d][1,3]dioxol-4-yloxy)-3-(4-(3-(4-ethylpiperazin-1-yl)propoxy)phenyl)-N,N-dimethylpropan-1-amine oxalate* (**11e**), Pale-white solid; m.p. 187–189 °C; yield 35.1%; 1H NMR (400 MHz, DMSO-*d*_6_) δ 7.32 (d, *J* = 8.2 Hz, 2H), 6.91 (d, *J* = 8.2 Hz, 2H), 6.65 (t, *J* = 8.1 Hz, 1H), 6.50 (dd, *J* = 14.4, 8.1 Hz, 2H), 5.98 (d, *J* = 12.5 Hz, 2H), 5.42 (dd, *J* = 8.3, 4.7 Hz, 1H), 4.05–3.95 (m, 2H), 3.33–3.11 (m, 2H), 3.08 (dt, *J* = 13.0, 7.4 Hz, 4H), 3.00–2.92 (m, 2H), 2.76 (s, 10H), 2.63 (t, *J* = 7.4 Hz, 2H), 2.33 (dt, *J* = 13.9, 7.9 Hz, 1H), 2.21–2.08 (m, 1H), 1.91 (p, *J* = 6.7 Hz, 2H), 1.16 (t, *J* = 7.2 Hz, 3H). ^13^C NMR (101 MHz, DMSO-*d*_6_) δ 158.74, 148.93, 141.66, 136.23, 132.44, 128.06, 122.25, 114.92, 111.73, 103.06, 101.35, 78.16, 65.78, 54.21, 53.80, 51.00, 50.23, 49.93, 42.65, 32.61, 25.74, 9.77. HRMS (ESI) *m*/*z*: calcd for: C_27_H_39_N_3_O_4_ [M + H]^+^: 470.3013; found: 470.3017.
*3-(benzo[d][1,3]dioxol-4-yloxy)-3-(4-(3-(4-isopropylpiperazin-1-yl)propoxy)phenyl)-N,N-dimethylpropan-1-amine oxalate* (**11f**), Pale-white solid; m.p. 160–162 °C; yield 49.3%; ^1^H NMR (400 MHz, DMSO-*d*_6_) δ 7.32 (d, *J* = 8.4 Hz, 2H), 6.91 (d, *J* = 8.4 Hz, 2H), 6.65 (t, *J* = 8.1 Hz, 1H), 6.55–6.50 (m, 1H), 6.50–6.43 (m, 1H), 5.97 (dd, *J* = 12.1, 1.0 Hz, 2H), 5.42 (dd, *J* = 8.4, 4.6 Hz, 1H), 3.98 (t, *J* = 6.2 Hz, 2H), 3.23 (ddt, *J* = 28.3, 11.8, 5.7 Hz, 2H), 3.12–3.07 (m, 4H), 2.75 (s, 7H), 2.63 (t, *J* = 7.3 Hz, 2H), 1.91 (p, *J* = 6.7 Hz, 2H), 1.18 (t, *J* = 7.2 Hz, 12H). ^13^C NMR (101 MHz, DMSO-d6) δ 164.66, 158.76, 148.94, 141.68, 132.45, 128.05, 122.24, 114.93, 111.74, 103.06, 101.34, 78.20, 65.85, 56.42, 54.21, 53.86, 50.29, 47.31, 45.88, 42.66, 32.63, 25.93, 17.18, 8.93. HRMS (ESI) *m*/*z*: calcd for: C_28_H_41_N_3_O_4_ [M + H]^+^: 484.3170; found: 484.3178.
*3-(benzo[d][1,3]dioxol-4-yloxy)-3-(4-(3-(hexahydrocyclopenta[c]pyrrol-2(1H)-yl)propoxy)phenyl)-N,N-dimethylpropan-1-amine oxalate* (**11g**), Pale-white solid; m.p. 110–112 °C; yield 44.1%; ^1^H NMR (400 MHz, DMSO-*d*_6_) δ 7.33 (d, *J* = 8.2 Hz, 2H), 6.91 (d, *J* = 8.2 Hz, 2H), 6.65 (t, *J* = 8.1 Hz, 1H), 6.50 (dd, *J* = 13.7, 8.1 Hz, 2H), 5.98 (d, *J* = 12.4 Hz, 2H), 5.43 (dd, *J* = 8.4, 4.6 Hz, 1H), 4.06–4.00 (m, 2H), 3.13 (dt, *J* = 48.0, 6.5 Hz, 4H), 2.74 (s, 10H), 2.33 (d, *J* = 11.1 Hz, 1H), 2.10 (dt, *J* = 22.8, 6.0 Hz, 3H), 1.71–1.43 (m, 6H), 1.18 (t, *J* = 7.1 Hz, 2H). ^13^C NMR (101 MHz, DMSO-*d*_6_) δ 158.49, 148.93, 141.68, 136.23, 132.68, 128.06, 122.24, 114.93, 111.73, 103.03, 101.35, 78.19, 65.35, 60.23, 58.75, 54.15, 50.94, 42.62, 41.16, 32.61, 31.20, 25.66, 25.03, 21.22, 14.54. HRMS (ESI) *m*/*z*: calcd for: C_28_H_38_N_2_O_4_ [M + H]^+^: 467.2904; found: 467.2920.
*3-(benzo[d][1,3]dioxol-4-yloxy)-N,N-dimethyl-3-(3-(3-(pyrrolidin-1-yl)propoxy)phenyl)propan-1-amine oxalate* (**11h**), Pale-white solid; m.p. 104–106 °C; yield 38.9%; ^1^H NMR (400 MHz, DMSO-*d*_6_) δ 7.28 (t, *J* = 7.9 Hz, 1H), 6.98 (d, *J* = 7.0 Hz, 2H), 6.86 (d, *J* = 8.2 Hz, 1H), 6.66 (t, *J* = 8.1 Hz, 1H), 6.52 (dd, *J* = 14.6, 8.1 Hz, 2H), 5.99 (d, *J* = 12.1 Hz, 2H), 5.45 (dd, *J* = 8.4, 4.1 Hz, 1H), 4.03 (q, *J* = 7.2 Hz, 2H), 3.32–3.09 (m, 8H), 2.74 (s, 6H), 2.34–2.06 (m, 4H), 1.92 (d, *J* = 6.5 Hz, 4H). ^13^C NMR (101 MHz, DMSO-*d*_6_) δ 158.86, 148.97, 142.43, 141.76, 136.15, 130.27, 122.32, 118.86, 114.46, 112.58, 111.42, 103.14, 101.43, 78.43, 65.23, 54.14, 53.39, 51.77, 42.69, 32.76, 25.73, 23.17. HRMS (ESI) *m*/*z*: calcd for: C_25_H_34_N_2_O_4_ [M + H]^+^: 427.2591; found: 427.2586.
*3-(benzo[d][1,3]dioxol-4-yloxy)-N,N-dimethyl-3-(3-(3-(piperidin-1-yl)propoxy)phenyl)propan-1-amine oxalate* (**11i**), Pale-white solid; m.p. 81–83 °C; yield 32.6%; ^1^H NMR(400 MHz, DMSO-*d*_6_) δ 7.28 (td, *J* = 7.8, 1.7 Hz, 1H), 7.01–6.94 (m, 2H), 6.86 (dd, *J* = 8.1, 2.4 Hz, 1H), 6.66 (t, *J* = 8.1 Hz, 1H), 6.57–6.47 (m, 2H), 5.99 (dd, *J* = 11.6, 1.0 Hz, 2H), 5.45 (dd, *J* = 8.4, 4.3 Hz, 1H), 4.09–3.95 (m, 2H), 3.14–3.09 (m, 4H), 2.73 (d, *J* = 1.5 Hz, 6H), 2.53–2.49 (m, 4H), 2.30 (dd, *J* = 19.6, 9.4 Hz, 1H), 2.22 –2.05 (m, 3H), 1.73 (p, *J* = 5.7 Hz, 4H), 1.60–1.45 (m, 2H). ^13^C NMR (101 MHz, DMSO-*d*_6_) δ 158.85, 148.96, 142.43, 141.76, 136.14, 130.27, 122.32, 118.85, 114.47, 112.58, 111.42, 103.13, 101.43, 78.44, 65.40, 54.15, 53.83, 52.48, 42.68, 32.76, 23.77, 22.93, 21.90. HRMS (ESI) *m*/*z*: calcd for: C_26_H_36_N_2_O_4_ [M + H]^+^: 441.2748; found: 441.2749.
*3-(benzo[d][1,3]dioxol-4-yloxy)-N,N-dimethyl-3-(3-(3-morpholinopropoxy)phenyl)propan-1-amine oxalate* (**11j**), Pale-white solid; m.p. 95–98 °C; yield 43.4%; ^1^H NMR (400 MHz, DMSO-*d*_6_) δ 7.28 (t, *J* = 8.0 Hz, 1H), 7.01–6.95 (m, 2H), 6.88– 6.82 (m, 1H), 6.67 (t, *J* = 8.1 Hz, 1H), 6.52 (dd, *J* = 16.1, 8.1 Hz, 2H), 5.99 (d, *J* = 11.0 Hz, 2H), 5.45 (dd, *J* = 8.4, 4.2 Hz, 1H), 4.01 (q, *J* = 7.3, 6.0 Hz, 2H), 3.77–3.69 (m, 4H), 3.17 (dtd, *J* = 32.2, 11.9, 4.6 Hz, 2H), 2.95–2.82 (m, 6H), 2.77 (s, 6H), 2.35–2.26 (m, 1H), 2.19 (d, *J* = 11.3 Hz, 1H), 2.06–1.97 (m, 2H). ^13^C NMR (101 MHz, Chloroform-*d*) δ 158.87, 148.88, 142.28, 141.64, 136.07, 130.19, 122.23, 118.71, 114.33, 112.54, 111.34, 103.06, 101.33, 78.29, 65.49, 64.58, 54.05, 52.12, 42.59, 32.61, 24.25. HRMS (ESI) *m*/*z*: calcd for: C_25_H_34_N_2_O_5_ [M + H]^+^: 443.2540; found: 443.2540.
*3-(benzo[d][1,3]dioxol-4-yloxy)-3-(3-(3-(4-isopropylpiperazin-1-yl)propoxy)phenyl)-N,N-dimethylpropan-1-amine oxalate* (**11k**), Pale-white solid; m.p. 185–186 °C; yield 42.5%; ^1^H NMR (400 MHz, DMSO-*d*_6_) δ 7.27 (t, J = 8.0 Hz, 1H), 6.97 (dd, *J* = 7.1, 1.5 Hz, 2H), 6.88–6.81 (m, 1H), 6.71–6.62 (m, 1H), 6.52 (ddd, *J* = 15.0, 8.2, 1.0 Hz, 2H), 5.99 (dd, *J* = 10.5, 1.0 Hz, 2H), 5.45 (dd, *J* = 8.5, 4.4 Hz, 1H), 4.00 (q, *J* = 6.1 Hz, 2H), 3.33–3.08 (m, 4H), 3.06 (d, *J* = 5.5 Hz, 3H), 2.76 (s, 9H), 2.65 (t, *J* = 7.4 Hz, 2H), 2.36–2.28 (m, 1H), 2.19 (d, *J* = 7.2 Hz, 1H), 1.92 (p, *J* = 6.7 Hz, 2H), 1.19 (d, *J* = 6.6 Hz, 7H). ^13^C NMR (101 MHz, Chloroform-*d*) δ 159.42, 153.04, 142.88, 134.85, 130.80, 128.29, 127.42, 126.70, 126.43, 126.00, 122.54, 121.07, 118.89, 114.33, 112.98, 107.94, 76.97, 66.10, 57.23, 54.75, 54.15, 50.37, 47.56, 43.10, 33.44, 26.00, 17.34. HRMS (ESI) *m*/*z*: calcd for: C_28_H_41_N_3_O_4_ [M + H]^+^: 484.3170; found: 484.3159.
*3-(benzo[d][1,3]dioxol-4-yloxy)-3-(4-(3-(dimethylamino)propoxy)phenyl)-N,N-dimethylpropan-1-amine oxalate* (**11l**), Pale-white solid; m.p. 121–122 °C; yield 35.1%;^1^H NMR (400 MHz, DMSO-*d*_6_) δ 7.33 (d, *J* = 8.2 Hz, 2H), 6.92 (d, *J* = 8.2 Hz, 2H), 6.65 (t, *J* = 8.1 Hz, 1H), 6.50 (dd, *J* = 12.2, 8.1 Hz, 2H), 5.98 (d, *J* = 12.0 Hz, 2H), 5.43 (dd, *J* = 8.3, 4.7 Hz, 1H), 4.01 (t, *J* = 6.0 Hz, 2H), 3.14 (q, *J* = 7.7 Hz, 3H), 3.05 (dt, *J* = 12.1, 6.1 Hz, 1H), 2.73 (d, *J* = 6.1 Hz, 12H), 2.33 (dt, *J* = 13.5, 7.4 Hz, 1H), 2.20–2.02 (m, 3H). ^13^C NMR (101 MHz, DMSO-*d*_6_) δ 158.50, 148.93, 141.69, 136.22, 132.72, 128.08, 122.25, 114.94, 111.73, 103.04, 101.35, 78.20, 65.33, 54.46, 54.16, 42.63 (d, *J* = 6.6 Hz), 32.64, 24.36. HRMS (ESI) *m*/*z*: calcd for: C_23_H_32_N_2_O_4_ [M + H]^+^: 401.2435; found:401.2529.
*3-(benzo[d][1,3]dioxol-4-yloxy)-3-(4-(3-(diethylamino)propoxy)phenyl)-N,N-dimethylpropan-1-amine oxalate* (**11m**), Pale-white solid; m.p. 98–100 °C; yield 30.3%; ^1^H NMR (400 MHz, DMSO-*d*_6_) δ 7.33 (d, *J* = 8.1 Hz, 2H), 6.92 (d, *J* = 8.0 Hz, 2H), 6.69–6.60 (m, 1H), 6.50 (dd, *J* = 12.2, 8.1 Hz, 2H), 5.98 (d, *J* = 12.2 Hz, 2H), 5.43 (dd, *J* = 8.3, 4.8 Hz, 1H), 4.05–4.00 (m, 2H), 3.16–3.03 (m, 8H), 2.73 (s, 6H), 2.37–2.26 (m, 1H), 2.11 (ddq, *J* = 22.7, 12.3, 6.4, 5.5 Hz, 3H), 1.18 (dd, *J* = 5.8, 3.3 Hz, 6H). ^13^C NMR (101 MHz, DMSO-*d*_6_) δ 158.48, 148.93, 141.69, 136.24, 132.72, 128.09, 122.25, 114.95, 111.76, 103.03, 101.36, 78.23, 65.30, 60.25, 54.15, 48.12, 46.45, 45.71, 42.62, 32.60, 23.45, 21.21, 14.53, 8.82 (d, *J* = 2.8 Hz). HRMS (ESI) *m*/*z*: calcd for: C_25_H_36_N_2_O_4_ [M + H]^+^: 429.2748; found: 429.2752.
*3-(benzo[d][1,3]dioxol-4-yloxy)-N,N-dimethyl-3-(4-(2-(piperidin-1-yl)ethoxy)phenyl)propan-1-amine oxalate* (**11n**), Pale-white solid; m.p. 116–118 °C; yield 33.2%;^1^H NMR (400 MHz, DMSO-*d*_6_) δ 7.35 (d, *J* = 8.3 Hz, 2H), 6.96 (d, *J* = 8.5 Hz, 2H), 6.65 (t, *J* = 8.1 Hz, 1H), 6.51 (dd, *J* = 11.1, 8.2 Hz, 2H), 5.98 (d, *J* = 12.3 Hz, 2H), 5.45 (dd, *J* = 8.3, 4.6 Hz, 1H), 4.32 (t, *J* = 4.9 Hz, 2H), 4.03 (q, *J* = 7.1 Hz, 2H), 3.41 (t, *J* = 4.9 Hz, 2H), 3.22 (dd, *J* = 11.8, 4.9 Hz, 1H), 3.10 (dt, *J* = 11.9, 6.1 Hz, 2H), 2.76 (s, 6H), 1.99 (s, 2H), 1.74 (*p*, *J* = 5.9 Hz, 5H), 1.18 (t, *J* = 7.1 Hz, 2H). ^13^C NMR (101 MHz, DMSO-*d*_6_) δ 164.86, 157.87, 157.77, 148.93, 141.64, 136.20, 133.20, 128.08, 122.26, 115.09, 111.68, 103.06, 101.35, 78.09, 62.68, 60.23, 55.15, 54.14, 52.95, 42.62, 32.63, 22.85, 21.67, 21.22, 14.54. HRMS (ESI) *m*/*z*: calcd for: C_25_H_34_N_2_O_4_ [M + H]^+^: 427.2591; found: 427.2587.
*3-(benzo[d][1,3]dioxol-4-yloxy)-N,N-dimethyl-3-(4-(4-(piperidin-1-yl)butoxy)phenyl)propan-1-amine oxalate* (**11o**), Pale-white solid; m.p. 169–171 °C; yield 42.9%; ^1^H NMR (400 MHz, DMSO-*d*_6_) δ 7.31–7.24 (m, 2H), 6.90–6.82 (m, 2H), 6.59 (d, *J* = 8.0 Hz, 1H), 6.46 (dd, *J* = 11.3, 7.9 Hz, 2H), 5.93 (d, *J* = 11.9 Hz, 2H), 5.37 (dd, *J* = 8.3, 4.8 Hz, 1H), 3.91 (t, *J* = 5.8 Hz, 2H), 3.13–2.86 (m, 8H), 2.65 (s, 6H), 2.26 (ddt, *J* = 13.4, 8.9, 5.3 Hz, 1H), 2.07 (ddt, *J* = 13.3, 10.3, 4.8 Hz, 1H), 1.69 (dp, *J* = 16.5, 5.1 Hz, 8H), 1.47 (s, 2H). ^13^C NMR (101 MHz, DMSO-*d*_6_) δ 165.45, 158.79, 149.01, 141.81, 136.29, 132.63, 128.12, 122.31, 115.00, 111.78, 103.08, 101.41, 78.31, 67.31, 56.12, 54.36, 52.46, 42.93, 32.95, 26.49, 23.04, 22.05, 20.79. HRMS (ESI) *m*/*z*: calcd for: C_27_H_38_N_2_O_4_ [M + H]^+^: 455.2904; found: 455.2906.
*3-(benzo[d][1,3]dioxol-4-yloxy)-N,N-dimethyl-3-(4-(3-(2-methylpiperidin-1-yl)propoxy)phenyl)propan-1-amine oxalate* (**11p**), Pale-white solid; m.p. 129–131 °C; yield 33.8%; ^1^H NMR (400 MHz, DMSO-*d*_6_) δ 7.34 (d, *J* = 8.2 Hz, 2H), 6.92 (d, *J* = 8.2 Hz, 2H), 6.67 (t, *J* = 8.1 Hz, 1H), 6.52 (dd, *J* = 12.0, 8.1 Hz, 2H), 5.98 (d, *J* = 12.2 Hz, 2H), 5.43 (dd, *J* = 8.3, 4.7 Hz, 1H), 4.07–3.99 (m, 2H), 3.36 (d, *J* = 11.7 Hz, 2H), 3.21–2.94 (m, 4H), 2.81 (t, *J* = 12.1 Hz, 2H), 2.69 (s, 6H), 2.31 (d, *J* = 11.8 Hz, 1H), 2.10 (dt, *J* = 16.0, 10.4 Hz, 3H), 1.75 (d, *J* = 13.7 Hz, 2H), 1.60 (d, *J* = 13.0 Hz, 1H), 1.38 (q, *J* = 12.4 Hz, 2H), 1.17 (d, *J* = 6.4 Hz, 3H). ^13^C NMR (101 MHz, DMSO-*d*_6_) δ 158.63, 148.61, 143.77, 136.84, 135.32, 128.78, 125.95, 123.95, 116.18, 114.60, 111.53, 104.55, 101.75, 79.32, 66.55, 56.81, 54.29, 53.76, 50.46, 44.98, 34.61, 33.87, 27.53, 25.78, 23.86, 18.36. HRMS (ESI) *m*/*z*: calcd for: C_27_H_38_N_2_O_4_ [M + H]^+^: 455.2904; found: 455.2908.
*3-(benzo[d][1,3]dioxol-4-yloxy)-N,N-dimethyl-3-(4-(3-(3-methylpiperidin-1-yl)propoxy)phenyl)propan-1-amine oxalate* (**11q**), Pale-white solid; m.p. 130–132 °C; yield 37.6%; ^1^H NMR (400 MHz, DMSO-*d*_6_) δ 7.33 (d, *J* = 8.2 Hz, 2H), 6.91 (d, *J* = 8.2 Hz, 2H), 6.65 (t, *J* = 8.1 Hz, 1H), 6.50 (dd, *J* = 12.0, 8.1 Hz, 2H), 5.98 (d, *J* = 12.2 Hz, 2H), 5.43 (dd, *J* = 8.3, 4.7 Hz, 1H), 4.04–4.00 (m, 2H), 3.36 (dd, *J* = 24.3, 11.6 Hz, 2H), 3.11 (tdd, *J* = 23.7, 12.0, 7.3 Hz, 4H), 2.73 (s, 8H), 2.46 (d, *J* = 11.7 Hz, 1H), 2.32 (d, *J* = 9.8 Hz, 1H), 2.12 (q, *J* = 7.2, 6.6 Hz, 3H), 1.89 (d, *J* = 17.1 Hz, 1H), 1.76–1.69 (m, 2H), 1.05 (dd, *J* = 11.8, 4.7 Hz, 1H), 0.89 (d, *J* = 6.5 Hz, 3H). ^13^C NMR (101 MHz, DMSO-*d*_6_) δ 158.49, 148.93, 141.69, 136.22, 132.72, 128.07, 122.24, 114.94, 111.71, 103.04, 101.34, 78.18, 65.46, 60.23, 57.97, 54.21, 53.86, 52.01, 42.73 (d, *J* = 2.2 Hz), 32.73, 30.52, 23.93, 22.69, 21.24, 19.06, 14.56. HRMS (ESI) *m*/*z*: calcd for: C_27_H_38_N_2_O_4_ [M + H]^+^: 455.2904; found: 455.2903.
*3-(benzo[d][1,3]dioxol-4-yloxy)-N,N-dimethyl-3-(4-(3-(4-methylpiperidin-1-yl)propoxy)phenyl)propan-1-amine oxalate* (**11r**), Pale-white solid; m.p. 126–128 °C; yield 42.8%; ^1^H NMR (400 MHz, DMSO-*d*_6_) δ 7.32 (d, *J* = 8.1 Hz, 2H), 6.91 (d, *J* = 8.1 Hz, 2H), 6.72–6.60 (m, 1H), 6.50 (dd, *J* = 12.9, 8.1 Hz, 2H), 5.97 (d, *J* = 12.0 Hz, 2H), 5.42 (t, *J* = 6.6 Hz, 1H), 4.07–3.99 (m, 2H), 3.36 (d, *J* = 11.7 Hz, 2H), 3.21–2.94 (m, 4H), 2.81 (t, *J* = 12.1 Hz, 2H), 2.69 (s, 6H), 2.31 (d, *J* = 11.8 Hz, 1H), 2.10 (dt, *J* = 16.0, 10.4 Hz, 3H), 1.75 (d, *J* = 13.7 Hz, 2H), 1.60 (d, *J* = 13.0 Hz, 1H), 1.38 (q, *J* = 12.4 Hz, 2H), 0.92 (d, *J* = 6.4 Hz, 3H). ^13^C NMR (101 MHz, DMSO-*d*_6_) δ 158.49, 148.93, 141.73, 136.22, 132.78, 128.06, 122.24, 114.93, 111.69, 103.02, 101.33, 78.21, 65.49, 60.23, 54.31, 53.65, 42.92, 32.94, 28.56, 24.18, 21.29 (d, *J* = 11.2 Hz), 14.56. HRMS (ESI) *m*/*z*: calcd for: C_27_H_38_N_2_O_4_ [M + H]^+^: 455.2904; found: 455.2912.
*3-(benzo[d][1,3]dioxol-4-yloxy)-3-(4-(3-(3,5-dimethylpiperidin-1-yl)propoxy)phenyl)-N,N-dimethylpropan-1-amine oxalate* (**11s**), Pale-white solid; m.p. 140–142 °C; yield 39.7%;^1^H NMR (400 MHz, DMSO-*d*_6_) δ 7.33 (d, *J* = 7.8 Hz, 2H), 6.91 (d, *J* = 8.0 Hz, 2H), 6.65 (t, *J* = 8.0 Hz, 1H), 6.50 (dd, *J* = 11.9, 8.1 Hz, 2H), 6.00– 5.90 (m, 2H), 5.44 (d, *J* = 6.8 Hz, 1H), 4.03 (dd, *J* = 12.6, 6.4 Hz, 3H), 3.36 (t, *J* = 8.3 Hz, 3H), 3.14 (s, 2H), 2.75 (d, *J* = 8.8 Hz, 6H), 2.42 (d, *J* = 12.1 Hz, 2H), 2.15 (s, 2H), 1.92 (s, 1H), 1.73 (d, *J* = 12.7 Hz, 1H), 1.21 (dt, *J* = 19.7, 4.7 Hz, 2H), 0.88 (d, *J* = 6.0 Hz, 7H), 0.78 (q, *J* = 12.3 Hz, 1H). ^13^C NMR (101 MHz, DMSO-*d*_6_) δ158.63, 148.60, 143.77, 137.03, 135.31, 127.96, 126.65, 123.95, 115.24, 111.53, 104.30, 101.75, 79.40, 66.45, 60.01, 54.25, 44.86, 39.01, 34.61, 29.12, 27.40, 18.64. HRMS (ESI) *m*/*z*: calcd for: C_28_H_40_N_2_O_4_ [M + H]^+^: 469.3061; found:469.3064.
*3-(benzo[d][1,3]dioxol-4-yloxy)-3-(4-(3-(4-fluoropiperidin-1-yl)propoxy)phenyl)-N,N-dimethylpropan-1-amine oxalate* (**11t**), Pale-white solid; m.p. 134–136 °C; yield 50.1%;^1^H NMR (400 MHz, DMSO-*d_6_*) δ 7.31 (t, *J* = 10.2 Hz, 2H), 6.91 (d, *J* = 8.4 Hz, 2H), 6.64 (d, *J* = 8.2 Hz, 1H), 6.50 (dd, J = 13.2, 8.1 Hz, 2H), 5.98 (d, *J* = 12.1 Hz, 2H), 5.42 (m, 1H), 5.12-4.89 (m,1H),4.02 (m, 2H),3.26–2.96 (m, 8H), 2.74 (s, 6H), 2.46 (d, J = 10.7 Hz, 1H), 2.26 (d, *J* = 6.7 Hz, 1H), 2.06 (p, *J* = 5.9 Hz, 2H), 1.73 –1.66 (m, 4H). ^13^C NMR (101 MHz, DMSO-*d*_6_) δ158.60, 148.51, 143.14, 136.07, 134.84, 128.51,126.07, 123.30, 115.74, 113.87, 111.71, 104.21, 100.91, 89.67, 87.88, 79.15, 66.16, 54.34, 50.01, 44.95, 34.71, 30.10, 27.28. HRMS (ESI) *m*/*z*: calcd for: C_26_H_35_FN_2_O_4_ [M + H]^+^: 459.2654; found: 459.2661.
*3-(benzo[d][1,3]dioxol-4-yloxy)-3-(4-(3-(4-chloropiperidin-1-yl)propoxy)phenyl)-N,N-dimethylpropan-1-amine oxalate* (**11u**), Pale-white solid; m.p. 145–146 °C; yield 39.4%; ^1^H NMR (400 MHz, DMSO-*d*_6_) δ 7.33 (d, *J* = 7.4 Hz, 2H), 6.92 (d, *J* = 7.5 Hz, 2H), 6.65 (t, *J* = 8.0 Hz, 1H), 6.51 (dd, *J* = 14.1, 8.1 Hz, 2H), 5.96 (t, *J* = 14.2 Hz, 2H), 5.46–5.37 (m, 1H), 3.99 (d, *J* = 2.7 Hz, 2H), 3.26 (ddd, *J* = 29.4, 14.6, 4.7 Hz, 4H), 3.17–2.97 (m, 6H), 2.76 (s, 6H), 2.35–2.24 (m, 3H), 2.13–2.06 (m, 2H), 2.04–1.96 (m, 2H). ^13^C NMR (101 MHz, DMSO-*d*_6_) δ 158.62, 148.60, 143.77, 136.91, 135.31, 127.98, 127.70, 123.95, 115.24, 111.72, 104.30, 101.74, 79.32, 66.24, 56.89, 54.47, 54.18, 50.87, 44.98, 34.61, 33.45, 27.28. HRMS (ESI) *m*/*z*: calcd for: C_26_H_35_ClN_2_O_4_ [M + H]^+^: 475.2358; found:475.2364.
*1-(3-(4-(1-(benzo[d][1,3]dioxol-4-yloxy)-3-(dimethylamino)propyl)phenoxy)propyl)piperidine-4-carbonitrile oxalate* (**11v**), Pale-white solid; m.p. 148–151 °C; yield 18.3%; ^1^H NMR (500 MHz, DMSO-*d*_6_) δ 7.33 (d, *J* = 8.2 Hz, 2H), 6.91 (d, *J* = 8.2 Hz, 2H), 6.65 (t, *J* = 8.1 Hz, 1H), 6.51 (dd, *J* = 14.6, 8.1 Hz, 2H), 5.98 (d, *J* = 12.4 Hz, 2H), 5.43 (t, *J* = 6.5 Hz, 1H), 3.99 (t, *J* = 6.1 Hz, 2H), 3.24–3.15 (m, 2H), 3.05 (td, *J* = 26.1, 23.9, 13.3 Hz, 6H), 2.76 (s, 6H), 2.36–2.29 (m, 1H), 2.20–2.01 (m, 5H), 1.96 (d, *J* = 11.2 Hz, 2H), 1.27–1.16 (m, 1H). ^13^C NMR (101 MHz, DMSO-*d*_6_) δ158.59, 148.65, 143.73, 137.13, 135.30, 127.71, 123.92, 121.09, 115.23, 111.43, 104.55, 101.65, 79.42, 66.14, 54.39, 52.42, 44.86, 34.61, 27.28, 26.49, 25.91. HRMS (ESI) *m*/*z*: calcd for: C_27_H_35_N_3_O_4_ [M + H]^+^: 466.2700; found: 466.2739.

### 3.4. Molecular Docking Study

All molecular docking calculations were performed with Glide SP Version 9.7 [36] (Schrödinger LLC, New York, NY, USA). X-ray structures of SERT (5I73) [37], NET (8XB2) [38], and histamine H_3_ receptor (7F61) [15] were prepared by Maestro (Schrödinger LLC). Centroids of original ligands of X-ray structures were defined as centers of boxes to generate the receptor grid. The van der Waals scaling factor and partial charge cutoff were set as 0.80 and 0.15 separately. The method of ligand sampling was flexible and intramolecular hydrogen bonds were rewarded. In total, 5000 poses per ligand were sampled for the initial phase of docking and 1000 poses per ligand were performed post-docking minimization. All docking results were visualized using Maestro (version. 2022-4, Schrödinger LLC).

### 3.5. Biological Studies

#### Ethics Statement

In this research, Chinese Kun Ming (KM) mice weighing 20 g (with a variance of ±2.0 g) and Sprague Dawley (SD) rats weighing 250 g (with a variance of ±5.0 g) were utilized as subjects for experimentation. The animals were maintained under uniform environmental conditions regarding lighting, temperature, and humidity, and they had unrestricted access to standard rodent food and water. They were randomly assigned to various experimental groups and each group was housed individually. All animal-related studies conducted in this project adhered to the regulations set forth for animal experimentation and received approval from the Ethics and Experimental Animal Committee of Jiangsu Nhwa Pharmaceutical Co., Ltd. (Xuzhou, China). For the procedural details of the biological studies, see the Supplementary Materials.

## 4. Conclusions

To sum up, we presented the synthesis and pharmacological assessment of a series of amphetamine derivatives as potential multitarget ADs in this study. Among these derivatives, compound **11b** exhibited high affinity for SERT, NET, and H_3_ receptors, and also had a favorable selectivity profile for nontarget receptors (H_1_ and α_1_) that are recognized to be related to the adverse effects of available ADs on the market. Molecular docking studies were meticulously carried out to firmly substantiate the experimental results, and during this process, the key interactions were precisely determined. In vivo animal models revealed that compound **11b** presented a lower MED in TST and FST in comparison with duloxetine, without a stimulating effect on the locomotor activity. Furthermore, compound **11b** displayed a low level of inhibition at the hERG channel, which is associated with no QT prolongation. Additionally, it had a higher threshold for acute toxicity than duloxetine. Finally, pharmacokinetic studies revealed that compound **11b** possessed a favorable drug-like pharmacokinetic profile. Therefore, we expect that compound **11b** might be valuable for the development of a new class of drugs for the treatment of depression.

## Figures and Tables

**Table 1:** Binding affinities for SERT, NET, and H_3_ receptor of compounds **11a**–**v** and reference antidepressant.

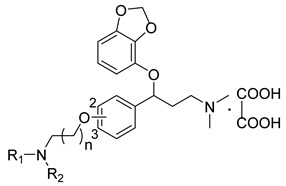				
**Compound**	**Structure**	**Receptor Affinity *K*_i_ ± SEM (nM) ^a^**		
		**SERT**	**NET**	**H_3_**
**11a**	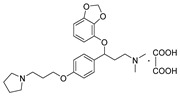	10.3 ± 0.9	19.2 ±2.3	4.2 ± 0.3
**11b**	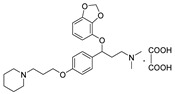	7.8 ± 0.9	10.5 ± 1.2	5.5 ± 0.4
**11c**	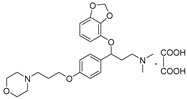	43.2 ± 3.8	32.6 ± 2.7	26.2 ± 2.1
**11d**	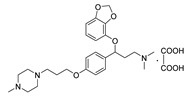	52.3 ± 2.8	86.8 ± 9.1	278.2 ± 30.1
**11e**	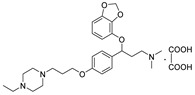	39.6 ± 3.3	66.9 ± 5.1	351.5 ± 32.3
**11f**	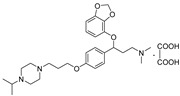	35.7 ± 3.1	57.9 ± 6.4	233.9 ± 21.8
**11g**	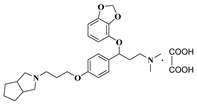	31.3 ± 4.0	48.2 ± 4.2	10.6 ± 1.7
**11h**	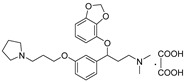	191.6 ± 18.8	144.2 ± 13.5	202.4 ± 23.6
**11i**	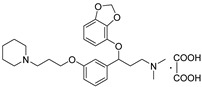	236.4 ± 26.1	105.4 ± 9.8	118.6 ± 10.5
**11j**	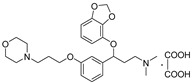	465.4 ± 39.8	152.9 ± 12.7	170.3 ± 15.9
**11k**	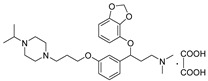	163.2 ± 17.6	111 ± 10.3	421.5 ± 39.4
**11l**	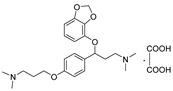	17.1 ± 1.6	114.9 ± 12.3	958.1 ± 87.6
**11m**	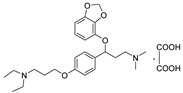	28.4 ± 11.3	85.2 ± 9.1	744.6 ± 68.3
**11n**	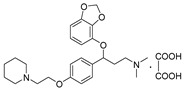	26.1 ± 2.3	37.3 ± 4.2	1021.5 ± 98.3
**11o**	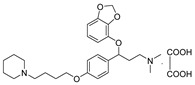	49.2 ± 4.8	64.6 ± 5.5	835.3 ± 79.1
**11p**	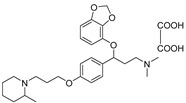	20.6 ± 1.9	35.4 ± 3.2	21.8 ± 2.3
**11q**	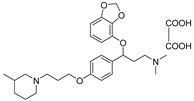	12.1 ± 1.6	20.7 ± 2.1	3.2 ± 0.2
**11r**	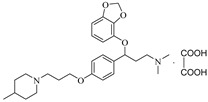	27.1 ± 1.6	38.3 ± 3.5	11.5 ± 1.0
**11s**	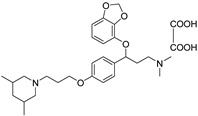	30.3 ± 2.6	25.1 ± 2.8	18.2 ± 1.9
**11t**	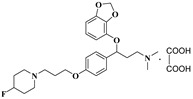	34.2 ± 2.8	42.5 ± 4.6	88.6 ± 9.1
**11u**	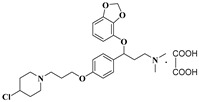	39.5 ± 4.0	50.2 ± 4.9	133.7 ± 3.0
**11v**	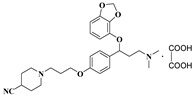	60.5 ± 5.7	48.6 ± 6.4	115.4 ± 12.8
Duloxetine		9.2 ± 1.1	31.5 ± 3.4	>1000
Pitolisant		>1000	>1000	0.58 ± 0.03

^a^ *K*_i_ values are taken from three experiments, expressed as means ± SEM.

**Table 2:** Binding affinity for α_1_ and H_1_ receptors of compounds **11a**, **11b**, **11q** and reference antidepressant.

Compound	Receptor Affinity *K*_i_ ± SEM (nM) ^a^		(IC_50_, nM)
	α_1_	H_1_	hERG
**11a**	428.4 ± 35.3	241.8 ± 30.5	819.1 ± 90.4
**11b**	1165.2 ± 125.8	896.9 ± 96.3	1000.8 ± 112.1
**11q**	306.7 ± 28.1	624.6 ± 57.7	779.6 ± 68.5
Duloxetine	1008.3 ± 97.8	1122.6 ± 121.5	1407.7 ± 153.8

^a^ *K*_i_ values are taken from three experiments, expressed as means ± SEM.

**Table 3:** Activities of compound **11b** and reference compounds to SERT, NET, and H_3_ receptors.

Receptor	Compd	Activation (10 μM, %) (*n* = 3)	EC_50_ (nM)	Inhibition (10 μM, %) (*n* = 3)	IC_50_ (nM)
SERT	Citalopram			101.2 ± 1.8	8.9
	**11b**	4.4 ± 0.3		98.5 ± 3.0	20.1
NET	Nisoxetine			99.8 ± 1.9	37.4
	**11b**	2.7 ± 0.2		100.1 ± 1.2	49.6
H_3_	Histamine	101.3 ± 2.7	14.1		
	Pitolisant			99.3 ± 2.5	2.45
	**11b**	5.3 ± 0.6		101.5 ± 2.0	0.72

**Table 4:** Pharmacokinetic profile of compound **11b** in tats (*n* = 6/group).

**Dose (mg/kg)**	**C** **_max_** **(ng/mL)**	**T** **_max_** **(h)**	**T** **_1/2_** **(h)**	**AUC_(0_** **–** **_t_** **_)_** **(ng** **·** **h/mL)**	AUC_(0_**–**_∞)_ (**ng·h/mL)**	**MRT_last_ (h)**	*F* (%)
2 (iv)	224.4	0.08	1.3	261.4	265.1	1.2	-
20 (po)	133.9	2.0	2.6	941.8	944.1	5.4	35.6

## Data Availability

Data are contained within the article and Supplementary Materials.

## Supplementary Materials

The following supporting information can be downloaded at: https://www.mdpi.com/1420-3049/29/22/5240/s1, Receptor Binding Assays; Intrinsic Activity Assessment; Selectivity of Compound **11b** for Additional Receptors; hERG Affinity; Acute Toxicity; Behavioral Studies; Pharmacokinetics Study; ^1^HNMR, ^13^C NMR, and HR-MS of Compound **11b**; ^1^H NMR of other Target Compounds. Refs. [21,26,39,40,41,42,43,44,45,46,47,48] are cited in Supplementary Materials file.
