# Supplementary material for: Amphetamine Derivatives as Potent Central Nervous System Multitarget SERT/NET/H3 Agents: Synthesis and Biological Evaluation
Source: Molecules. 2024 Nov 6;29(22):5240. doi: 10.3390/molecules29225240 (PMC11870037; doi:10.3390/molecules29225240)
Supplement: Supplementary file 1 [file molecules-29-05240-s001.zip › molecules-3279156-supplementary.pdf]

Supplementary Material

Amphetamine Derivatives as Potent CNS Multi-target SERT/NET/H_3 agents: Synthesis and Biological Evaluation

Quxiang Li[1], Lili Ren[1], Dongli Wang[2], Junyong Luo[1], Changda Xu[2], Jian Feng[2], Yufan Qiu[3], Xiangqing Xu [2,]*, Guoguang Chen [1,]*

[1] School of Pharmacy, Nanjing Tech University, 30th South Puzhu Road, Nanjing 211816, China; liquxiang@163.com (Q.L.); renlili@njtech.edu.cn (R.L.); junyongluo@163.com (J.L.).

[2] Institute of Pharmaceutical Research, Jiangsu Nhwa Pharmaceutical Co., Ltd. & Jiangsu Key Laboratory of Central Nervous System Drug Research and Development, Xuzhou 221116, China; wangdongli@nhwa-group.com (D.W.); xuchangda@nhwa-group.com (C.X.); fengjian@nhwa-group.com(J.F.).

[3] Pharmaron Beijing Co., Ltd., 6 Taihe Road, BDA, Beijing 100176, China; yufan.qiu@pharmaron-bj.com (Y.Q.).

[*] Correspondence: xuxiangqing@nhwa-group.com (X.X.); ggchen@njtech.edu.cn (G.C).

Table of Contents:

1. Receptor Binding Assays S2-S4
2. Intrinsic Activity Assessment S4-S5
3. Selectivity of Compound **11b** for Additional Receptors S5-S10
4. hERG Affinity S11
5. Acute Toxicity S11
6. Behavioral Studies S11-S12
7. Pharmacokinetics Study S12
8. ^{1}HNMR, ^{13}C NMR and HR-MS of Compound **11b** S13- S14
9. ^{1}H NMR of other Target Compounds S14- S24
10. References S25

1. Receptor Binding Assays

Materials

The following specific radioligands and tissue sources were used: (a) the SERT receptor, [^{3}H]-paroxetine, rat cerebral cortex; (b) the NET receptor, [^{3}H]nisoxetine, from hippocampal membrane; (c) Histamine H_3 receptor, [^{3}H]-methylhistamine, rat cerebral cortex; (d) the histamine H_1 receptor, [^{3}H]pyrilamine, from guinea pig cerebellum; (e) the adrenergic α_1 receptor, [^{3}H]prazosin, from rat cerebral cortex.

General Procedures for the Binding Assays

All new compounds were dissolved in a 50% (v/v) solution of DMSO, achieving a compound concentration of 2 × 10^{-3} M. To prepare a dilution to the initial concentration of the new compounds at 2 × 10^{-4} M, the solution contained 5% DMSO. In the SERT receptor binding assays, the total binding (TB) was measured in the presence of the radiolabeled ligand [^{3}H]imipramine. Nonspecific binding (NB) was ascertained in the presence of unlabeled imipramine, and compound binding (CB) was assessed in the presence of both the radiolabeled ligand [^{3}H]imipramine and the compound under investigation. The specific binding (SB) for each condition was calculated by subtracting the nonspecific binding (NB) from the total binding (TB) at a given concentration of the radioligand.

Each percentage of inhibition (%) was calculated as follows: percentage of inhibition (%) = [(TB - CB)/(TB - NB)] × 100.

Control binding experiments, which included a 0.25% (v/v) concentration of DMSO, were conducted and showed that DMSO did not exert any influence. Each compound was evaluated in triplicate at a range of concentrations that spanned six orders of magnitude, from 10^{-5} M to 10^{-10} M. The IC_{50} values, indicative of the concentration at which 50% inhibition occurred, were ascertained through a nonlinear regression analysis, fitting the data to the Hill equation curve. The K_i values, which represent the inhibition constant, were computed using the formula established by Cheng and Prussoff: $K_i = IC_{50}/(1+C/K_d)$. In this equation, C denotes the concentration of the competing (hot) ligand, and K_d is the dissociation constant of the respective radiolabeled ligand. The average K_i values along with their standard error of the mean (SEM) were calculated from a minimum of three separate experiments.

SERT binding assay [1]

The rat's cerebral cortex was processed by homogenization in twenty volumes of an ice-cold Tris-HCl buffer solution at a concentration of 50 mM and a pH of 7.7, which also contained 150 mM of sodium chloride (NaCl) and 5 mM of potassium chloride (KCl). This was accomplished using an ULTRA TURAX homogenizer, followed by centrifugation at 20,000g for 20 minutes. The resulting pellet was then re-suspended in an equal volume of the same buffer and centrifuged again for 20 minutes. The final pellet was subsequently re-suspended in a volume of Tris-HCl buffer that was fifty times its original volume. For the total binding assay, each tube received 900 μL of the tissue suspension, supplemented with 50 μL of a 1 nM solution of [3H]-paroxetine and 50 μL of the Tris-HCl buffer. In the nonspecific binding assay, each tube was prepared with 900 μL of the tissue suspension, 50 μL of the 1 nM [^{3}H]-paroxetine, and 50 μL of a 10 μM paroxetine solution to block nonspecific binding sites. For the specific

binding assay, each tube contained 900 μL of the tissue suspension, 50 μL of a 0.6 nM [^{3}H]-paroxetine solution, and 50 μL of either a new compound or a reference drug solution at various concentrations ranging from 10^{-5} mol to 10^{-10} mol. Following the preparation, the samples were incubated at a temperature of 37°C for 10 minutes. After incubation, the samples were quickly filtered under vacuum through Whatman GF/B glass fiber filters. The filtrates were then washed twice with 5 mL of cold buffer and transferred to scintillation vials. To each vial, 1.0 mL of scintillation fluid was added, and the radioactivity associated with the bound substances was quantified using a PE Microbeta 2450 liquid scintillation counter.

NET binding assay [1]

The hippocampal tissue from a rat was processed by homogenization in twenty volumes of a chilled 50 mM phosphate buffer solution with a pH of 7.4, using an ULTRA TURAX homogenizer. The homogenate was subjected to two rounds of centrifugation, each lasting for 10 minutes at 35,000g, with the pellet being re-suspended in fresh buffer between spins. The final pellet was then re-suspended in a membrane buffer composed of 20 mM HEPES, 145 mM NaCl, and 5 mM KCl, adjusted to a pH of 7.4. For the total binding assay, each tube was prepared with 900 μL of the membrane suspension, 50 μL of a 1 nM solution of [^{3}H]-nisoxetine, and 50 μL of the membrane buffer. In the nonspecific binding assay, each tube contained 900 μL of the membrane suspension, 50 μL of the 1 nM [^{3}H]-nisoxetine, and 50 μL of a 1 μM desipramine solution to account for nonspecific interactions. For the specific binding assay, each tube was filled with 900 μL of the membrane suspension, 50 μL of the 1 nM [^{3}H]-nisoxetine, and 50 μL of either a new compound or a reference drug solution at varying concentrations ranging from 10^{-5} mol to 10^{-10} mol. Following preparation, the samples were incubated at a temperature of 30°C for a duration of 8 hours. Post-incubation, the samples were quickly filtered under vacuum through Whatman GF/B glass fiber filters. The filtrates were then washed twice with 5 mL of cold buffer solution and transferred to scintillation vials. To each vial, 3.0 mL of scintillation fluid was added, and the radioactivity of the bound substances was quantified using a PE Microbeta 2450 liquid scintillation counter.

Histamine H_3 receptor [2]

Rat cerebral cortex was homogenized in 20 volumes of ice-cold membrane buffer (5 mM Tris-HCl, pH 7.7; 5 mM EDTA and protease inhibitor) using an ULTRA TURAX homogenizer, and centrifuged at 40000g for 30min at 4 °C. The resulting membrane pellets were washed by resuspension in membrane buffer (50 mM Tris-HCl, pH 7.7; 0.6 mM EDTA, 5 mM $MgCl_2$ and protease inhibitors) and centrifuged for 20 min. The final pellet was resuspended in 50volumes of the Tris-HCl buffer. For total binding, to each assay tube was added 900μL of the tissue suspension, 50 μL of 1 nM [^{3}H]-methylhistamine, and 50 μL of membrane buffer. For nonspecific binding, to each assay tube was added 900 μL of the tissue suspension, 50 μL of 1 nM [^{3}H]-methylhistamine, and 50 μL of 10 μM thioperamide. For specific binding, to each assay tube was added 900 μL of the tissuesuspension, 50 μL of 1 nM [^{3}H]-methylhistamine, 50 μL of new compound solution in various concentrations (10^{-5} mol to 10^{-10} mol). The tubes were incubated at 25 °C for 30 min. The incubation was followed by rapid vacuum filtration through Whatman GF/B glass filters, and the filtrates were washed twice with 5 mL of cold buffer and transferred to scintillation vials. Scintillation fluid (1.0 mL) was added, and the radioactivity

bound was measured using a PE Microbeta 2450 liquid scintillation counter.

Histamine H_1 receptor binding assay [1]

The guinea pig cerebellum was homogenized with an ULTRA TURAX homogenizer in 20 vol of ice-cold 50 mM phosphate buffer (pH 7.7), and then centrifuged twice for 10 min at 50000g, with the pellet being resuspended in fresh buffer. The final pellet was resuspended in phosphate buffer. In the case of total binding, 900 μL of membranes, 50 μL of 1 nM [^{3}H]pyrilamine (20.0 Ci/mmol; PerkinElmer Life Sciences, Boston, MA, USA), and 50 μL of phosphate buffer were added to each assay tube. When it came to nonspecific binding, 900 μL of membranes, 50 μL of [^{3}H]pyrilamine, and 50 μL of 1 mM promethazine were added to each assay tube. For compound binding, 900 μL of membranes, 50 μL of [^{3}H]pyrilamine, and 50 μL of new compounds or reference drug were added to each assay tube. The tubes were incubated at 30 °C for 60 min. Then, a rapid vacuum filtration was performed through Whatman GF/B glass filters, and the filtrates were washed twice with 5 mL of cold buffer and transferred to scintillation vials. Scintillation fluid (1.0 mL) was added, and the radioactivity that was bound was measured using a PE Microbeta 2450 liquid scintillation counter.

Adrenergic α_1 receptor binding assay [1]

Rat cerebral cortex was homogenized with an ULTRA TURAX homogeniser in 20 vol of ice-cold Tris-HCl buffer containing 5 mM EDTA (50 mM, pH 7.4) and was centrifuged at 44000 g for 20 min at 4 °C. The resulting pellet was resuspended in the same volume of the buffer and centrifuged for 20 min. The final pellet was resuspended in 50 vol of the Tris-HCl buffer. For total binding, 900 mL of the tissue suspension, 50 mL of 1 nM [^{3}H]prazosin (85.4 Ci/mmol; PerkinElmer Life Sciences, Boston, MA, USA), and 50 mL of Tris-HCl buffer were added to each assay tube. For nonspecific binding, 900 mL of the tissue suspension, 50 mL of 1 nM [^{3}H]prazosin, and 50 mL of 10 mM prazosin were added to each assay tube. For compound binding, 900 mL of the tissue suspension, 50 mL of [^{3}H]prazosin, and 50 mL of new compounds or reference drug were added to each assay tube. The tubes were incubated at 25 °C for 60 min. Then, rapid vacuum filtration was conducted through Whatman GF/B glass filters, and the filtrates were washed twice with 5 mL of cold buffer and transferred to scintillation vials. Scintillation fluid (1.0 mL) was added, and the radioactivity bound was measured using a PE Microbeta 2450 liquid scintillation counter.

2. Intrinsic Activity Assessment

Monoamine uptake transporter assay [3]

Using membrane preparations of rat cerebral cortex, the uptake of [^{3}H]5-HT by SERT or [^{3}H]NE by NET was carried out. Crude synaptosomes were incubated in a Krebs bicarbonate buffer solution containing [^{3}H]5-HT (20 nM), [^{3}H]NE (20 nM) and various concentrations of test compounds (10^{-1}–10^{-4} nM) at 37°C for 10 minutes. The nonspecific uptake to SERT and NET was determined respectively with 10 mM paroxetine, 10 mM desipramine, and 10 mM nomifensine. All experiments were executed in triplicate from three independent tests.

H_3 receptor intrinsic activity Assay [4]

Antagonist potency was determined according to literature, by measuring inhibition of RAMH-induced [^{35}S]GTPγS binding in recombinant hH_3R membranes as previously. The concentration of RAMH was 100 nM, which produces approximately 80% of maximum RAMH-induced signal. Inverse agonist potency was conducted by measuring inhibition of basal [^{35}S]GTPγS binding in recombinant hH_3 Rmembranes. Test compound or vehicle was added to the wells, followed by [^{35}S]GTPγS to a final concentration of 0.2 nM. Nonspecific binding was determined in the presence of 10 μM unlabeled GTPγS. The control agonist signal was determined in wells containing vehicle in place of the test compound, and the basal signal was determined in wells containing vehicle in place of both diluted compound and the RAMH challenge. Ciproxifan decreased basal [^{35}S]GTPγS binding in a concentration-dependent manner in the recombinant systems consistent with known inverse agonist activity of the compound **11b**.

3. Selectivity of Compound 11b for Additional Receptors

Materials

The following specific radioligands and tissue sources were used: (1) Dopamine transporter, ^{3}[H]-WIN35,428, rat striatum; (2) Dopamine D_1 receptor, ^{3}[H]-SCH23390, rat striatum; (3) Dopamine D_2 receptor, [^{3}H]spiperone, rat striatum; (4) Dopamine D_3 receptor, [^{3}H] 7-OH-DPAT, rat olfactory tubercle; (5) Serotonin 5-HT_{2A} receptor, [^{3}H]Ketanserin, rat brain cortex; (6) Serotonin 5-HT_{2C} receptor, [^{3}H]mesulergine, rat brain cortex; (7) Adrenergic α_2 receptor, [^{3}H]-rauwolscine, rat cerebral cortex; (8) Histamine H_2 receptor, [^{3}H]tiotidine, Sf9 cells and membrane; (9) Histamine H_4 receptor, [^{3}H]N^{a}-Methylhistamine, Sf9 cells and membrane; (10) M_1-M_5 receptor, [^{3}H]NMS, lymphocyte suspension and sections; (11) σ_1 receptor, [^{3}H]-(+)-pentazocine, male Dunkin Hartley guinea pig brain membrane; (12) σ_2 receptor, [^{3}H]-dio-tolylguanidine, male Dunkin-Hartley guinea pig brain membrane. (13) NMDA receptor, [^{3}H]-MK-801 (NET 972; Perkin-Elmer), and rat cerebral cortex.

Dopamine transporter [5]

Rat striatum was homogenized in 20 vol of ice-cold 50mM Tris-HCl buffer (pH 7.7) using an ULTRA TURAX homogeniser, and centrifuged twice for 10 min at 48,000 g with resuspension of the pellet in fresh buffer. The final pellet was resuspended in 50 mM ice-cold Tris-HCl containing 120 mM NaCl, 5 mM KCl, 2 mM $CaCl_2$, 1 mM $MgCl_2$, 0.1% ascorbic acid and 5 mM pargyline. For total binding, to each assay tube was added 900 μL of membranes, 50 μL of 0.5 nM [^{3}H]-WIN35,428, and 50 μL of membrane buffer. For nonspecific binding, to each assay tube was added 900 μL of membranes, 50 μL of 0.5 nM [^{3}H]-WIN35,428, and 50 μL of 1 μM Nomifensine. For specific binding, to each assay tube was added 900 μL of membranes, 50 μL of 0.5 nM [^{3}H]-WIN35,428, 50 μL of compound **11b** solution in various concentrations (10^{-5} mol to 10^{-10} mol). The tubes were incubated at 30°C for 8h. The incubation was followed by rapid vacuum filtration through Whatman GF/B glass filters, and the filtrates were washed twicewith 5 mL of cold buffer and transferred to scintillation vials. Scintillation fluid (1.0mL) was added, and the radioactivity bound was measured using a PE Microbeta 2450 liquid scintillation counter.

Dopamine D_1 receptor [6]

Rat striatum was homogenized in 20 vol of ice-cold 50mM Tris-HCl buffer (pH 7.4) using an ULTRA TURAX homogeniser, and centrifuged twice for 10 min at 48,000 g with resuspension of the pellet in fresh buffer. The final pellet was resuspended in 50 mM ice-cold Tris-HCl containing 120 mM NaCl, 5 mM KCl, 2 mM $CaCl_2$, 1 mM $MgCl_2$, 0.1% ascorbic acid and 1mM pargyline. For total binding, to each assay tube was added 900μL of the tissue suspension, 50 μL of 2 nM ^{3}[H]-SCH23390, and 50 μL of membrane buffer. For nonspecific binding, to each assay tube was added 900 μL of the tissue suspension, 50 μL of 2 nM ^{3}[H]-SCH23390, and 50 μL of 1 μM Butaclamol. For specific binding, to each assay tube was added 900 μL of the tissuesuspension, 50 μL of 2 nM ^{3}[H]-SCH23390, 50 μL of compound **(*R*)-13j** solution in various concentrations (10^{-5} mol to 10^{-10} mol). The tubes were incubated at 37 °C for 20 min. The incubation was followed by rapid vacuum filtration through Whatman GF/B glass filters, and the filtrates were washed twice with 5 mL of cold buffer and transferred to scintillation vials. Scintillation fluid (1.0 mL) was added, and the radioactivity bound was measured using a PE Microbeta 2450 liquid scintillation counter.

Dopaminergic D_2 Receptor [1]

Rat striatum was homogenized in 20 vol of ice-cold 50 mM Tris-HCl buffer (pH 7.7) using an ULTRA TURAX homogeniser, and centrifuged twice for 10 min at 48,000 g with resuspension of the pellet in fresh buffer. The final pellet was resuspended in 50 mM ice-cold Tris-HCl containing 120 mM NaCl, 5 mM KCl, 2 mM $CaCl_2$, 1 mM $MgCl_2$, 0.1% ascorbic acid and 5 mM pargyline. Total binding each assay tube was added 900 μL of the tissue suspension, 50 μL of 0.5 nM [^{3}H]spiperone (16.2 Ci/mmol; PerkinElmer Life Sciences, Boston, MA, USA), 50 mL Tris-HCl buffer containing 120 mM NaCl, 5 mM KCl, 2 mM $CaCl_2$, 1 mM $MgCl_2$, 0.1% ascorbic acid and 5 mM pargyline. Non-specific binding each assay tube was added 900 mL of the tissue suspension, 50 μL of 0.5 nM [^{3}H]spiperone, 50 μL of 10 mM (+)-butaclamol. Specific binding each assay tube was added 900 μL of the tissue suspension, 50 mL of 0.5 nM [^{3}H]spiperone, 50 μL of compound **11b** solution in various concentrations (10^{-5} mol to 10^{-10} mol). The tubes were incubated at 37 °C for 15 min. The incubation was followed by a rapid vacuum filtration through Whatman GF/B glass filters, and the filtrates were washed twice with 5 mL cold buffer and transferred to scintillation vials. Scintillation fluid (3.0 mL) was added and the radioactivity bound was measured using a PE Microbeta 2450 liquid scintillation counter.

Dopaminergic D_3 Receptor [7]

A rat olfactory tubercle was homogenized in 20 volumes of ice-cold 50 mM Hepes Na (pH 7.5) using an ULTRA TURAX homogenizer and centrifuged twice for 10 min at 48,000 g with resuspension of the pellet in fresh buffer. The final pellet was resuspended in 50 mM Hepes Na, pH 7.5, containing 1 mM EDTA, 0.005% ascorbic acid, 0.1% albumin, and 200 nM eliprodil. For total binding, to each assay tube was added 900 μL of membranes, 50 μL of 0.6 nM [^{3}H] 7-OH-DPAT (50 Ci/mmol; Perkin-Elmer Life Sciences, Boston, MA, USA), and 50 μL of 50 mM Hepes Na, pH 7.5, containing 1 mM EDTA, 0.005% ascorbic acid, 0.1% albumin, and 200 nM eliprodil. For nonspecific binding, to each assay tube was added 900 μL of membranes, 50 μL of [^{3}H] 7-OH-DPAT (50 Ci/mmol; Perkin-Elmer Life Sciences, Boston, MA, USA), and 50 μL of 1 μM dopamine. For specific binding, to each assay tube was added 900 μL of membranes, 50 μL of [^{3}H] 7-OH-DPAT (50 Ci/mmol; Perkin-Elmer Life Sciences, Boston, MA, USA), 50 μL of

compound **11b**, or reference drug. The tubes were incubated at 25 °C for 60 min. The incubation was followed by rapid vacuum filtration through Whatman GF/B glass filters, and the filtrates were washed twice with 5 mL of cold buffer and transferred to scintillation vials. Scintillation fluid (1.0 mL) was added, and the radioactivity bound was measured using a PE Microbeta 2450 liquid scintillation counter.

5-HT$_{2A}$ receptor [1]

The cerebral cortex of a rat was finely homogenized using an ULTRA TURAX homogenizer in 20 volumes of chilled Tris-HCl buffer at a concentration of 50 mM and a pH level of 7.7. This mixture was then centrifuged at a force of 32,000 g for a period of 20 minutes. The pellet that was obtained was subsequently re-suspended in an equivalent volume of the same buffer and centrifuged again for 20 minutes. The final pellet was then re-suspended in a volume of Tris-HCl buffer that was 50 times its own volume. For the total binding assays, each tube was prepared with 900 μL of the homogenized tissue suspension, 50 μL of a 0.6 nM [^{3}H]ketanserin solution with a radioactivity of 60.0 Ci/mmol, and 50 μL of Tris-HCl buffer. This [^{3}H]ketanserin was supplied by PerkinElmer Life Sciences, located in Boston, MA, USA. In the nonspecific binding assays, each tube contained 900 μL of the tissue suspension, 50 μL of the 0.6 nM [^{3}H]ketanserin, and 50 μL of a 10 μM methisergide solution to account for nonspecific interactions. For the specific binding assays, each tube was filled with 900 mL of the tissue suspension, 50 μL of the 0.6 nM [^{3}H]ketanserin, and 150 mL of compound **11b** or a reference drug to be tested. Following preparation, the samples in the tubes were incubated at a temperature of 37°C for 15 minutes. After incubation, the samples underwent rapid vacuum filtration using Whatman GF/B glass fiber filters. The resulting filtrates were then washed twice with 5 mL of cold buffer solution and subsequently transferred to scintillation vials. To each vial, 1.0 mL of scintillation fluid was added to measure the radioactivity of the bound substances using a PE Microbeta 2450 liquid scintillation counter.

5-HT$_{2C}$ receptor [1]

The cerebral cortex of a rat was processed by homogenization in a solution of ice-cold Tris-HCl buffer at a concentration of 50 mM and a pH of 7.7, using an ULTRA TURAX homogenizer. The mixture was then subjected to centrifugation at 32,000 g for 20 minutes. The pellet that formed was re-suspended in an equal volume of the same buffer and centrifuged again for another 20 minutes. The final pellet obtained was then re-suspended in a volume of Tris-HCl buffer equivalent to 50 times its volume. For the binding assays, each tube received a total volume of 900 μL of the tissue suspension, along with 50 μL of a 1 nM solution of [^{3}H]mesulergine, which has a specific activity of 85.4 Ci/mmol and was sourced from PerkinElmer Life Sciences in Boston, MA, USA, and 50 μL of the Tris-HCl buffer for total binding assays. For the non-specific binding assays, each tube was prepared with 900 μL of tissue suspension, 50 μL of the 1 nM [^{3}H]mesulergine, and 50 μL of a 10 μM mianserin solution. In the case of specific binding assays, each tube was filled with 900 μL of tissue suspension, 50 μL of the 1 nM [^{3}H]mesulergine, and 50 μL of compound **11b** or a reference drug. Following the preparation, the assays were incubated at a temperature of 37°C for a duration of 15 minutes. Post-incubation, the samples were quickly filtered under vacuum through Whatman GF/B glass fiber filters. The filtrates were then washed twice with 5 mL of cold buffer and

subsequently transferred to scintillation vials. To each vial, 1.0 mL of scintillation fluid was added, and the radioactivity associated with the bound material was quantified using a PE Microbeta 2450 liquid scintillation counter.

Adrenergic α_2 receptor [7]

Rat cerebral cortex was homogenized in 20 volumes of ice-cold Tris-HCl buffer containing 5 mM EDTA (50 mM, pH 7.7) using an ULTRATURAX homogenizer and centrifuged at 44000g for 20 min at 4 °C. The resulting pellet was resuspended in the same quantity of the buffer centrifuged for 20 min. The final pellet was resuspended in 50 volumes of the Tris-HCl buffer. For total binding, to each assay tube was added 900 μL of the tissue suspension, 50 μL of 1 nM [^{3}H]-rauwolscine, and 50 μL of Tris-HCl buffer. For nonspecific binding, to each assay tube was added 900 μL of the tissue suspension, 50 μL of 1 nM [^{3}H]-rauwolscine, and 50 μL of 10 μM rauwolscine. For specific binding, to each assay tube was added 900 μL of the tissue suspension, 50 μL of 1 nM [^{3}H]-rauwolscine, 50 μL of compound **11b** solution in various concentrations (10^{-5} mol to 10^{-10} mol). The tubes were incubated at 25 °C for 60 min. The incubation was followed by rapid vacuum filtration through Whatman GF/B glass filters, and the filtrates were washed twice with 5 mL of cold buffer and transferred to scintillation vials. Scintillation fluid (1.0 mL) was added, and the radioactivity bound was measured using a PE Microbeta 2450 liquid scintillation counter.

Histamine H_2, H_4 receptor [8]

[^{3}H]tiotidine, [^{3}H]N^a-Methylhistamine were from Hartmann analytic (Braunschweig, Germany). Sf9 membranes were thawed and sedimented by centrifugation at 13,000 g and 4°C for 10 min. The membranes were resuspended in cold (4°C) binding buffer (BB: 12.5 mM MgCl2, 1 mM EDTA and 75 mM Tris/HCl, pH 7.4) so that the final concentration was 2-6 μg of protein per 1 μL BB. Experiments were performed in 96-well plates (PP microplates 96 well, Greiner Bio-One, Frickenhausen, Germany) in a total volume of 100 μL containing 20-60 μg of membrane, 0.05% BSA, the respective radioligand and the investigated ligands at various concentrations (dissolved in water). During the incubation period of 60 min at room temperature, the plates were shaken at 250 rpm. Afterwards, bound radioligand was separated from free radioligand by filtration through, PEI covered, GF/C filters using a 96-well Brandel harvester (Brandel Inc., Unterföhring, Germany). After two washing steps with binding buffer, filter pieces were punched and transferred into untapped 96-well sample plates 1450-401 (Perkin Elmer, Rodgau, Germany). Each well was supplemented with 200 μL of scintillation cocktail (Rotiscint Eco plus, Roth, Karlsruhe, Germany) and incubated in the dark for 12 h. Radioactivity was measured. Radioactivity was measured with a MicroBeta2 1450 scintillation counter (PerkinElmer, Rodgau, Germany).

M_1-M_5 receptor [9]

In experiments for muscarinic cholinergic receptor subtype characterization, peripheral blood lymphocytes were used at a concentration of 1×10^6 cells in a 300-μl volume. For labeling muscarinic M_1 receptor, peripheral blood lymphocytes and brain sections were incubated with 0.3 nM [^{3}H]pirenzepine for 60 min at 25°C in Tris-$MgCl_2$ buffer (Flynn et al., 1997; Reever et al., 1997). For labeling M_2 muscarinic receptor, lymphocyte suspension and sections were pre-

incubated in a phosphate-$MgCl_2$ buffer containing 30 μg/ml of mamba venom for 30 min at 20°C, followed by incubation with 4-diphenylacetoxy *N*-methylpiperidine methiodide (4-DAMP) (10 nM) plus pirenzepine (0.3 μM) for 30 min. Pellets or sections were then incubated with 0.5 nM [^{3}H]NMS for 5 min (Flynn et al., 1997; Reever et al., 1997). For labeling M_3 muscarinic cholinergic receptor, lymphocyte suspension and sections were pre-incubated in a phosphate-$MgCl_2$ buffer containing 30 μg/ml of mamba venom for 30 min at 20°C, followed by incubation with 30 nM 11-((4-(4-(diethylamino)butyl)-1-piperidinyl) acetyl)-5,11-dihydro-6H-pyrido(2,3-b)(1,4)benzodiazepine-6-one-HCl (AQ-RA 741) for 30 min. Pellets or sections were then exposed to 0.5 nM. For labeling M_4 muscarinic cholinergic receptor, lymphocyte suspension and sections were pre-incubated for 30 min at 20°C in a phosphate-$MgCl_2$ buffer containing 10 nM S(+)-3-phenyl-1'-(phenylmethyl)-[3,4'-bipiperidine]-2,6-dione-HCl (S(+)-dexetimide-HCl), followed by incubation with 200 nM 11-((2-(diethylamino)methyl)-1-piperidinyl)acetyl)-5,11-dihydro-6H-pyrido-(2,3-b)(1,4)benzodiazepine-6-one-HCl (AF-DX 116) (200 nM) plus 150 nM pirenzepine for 30 min at 20°C. After this incubation, pellets or sections were exposed to a 0.5-nM concentration of [^{3}H]NMS for 15 min followed by a 5-min dissociation in the presence of 1 μM atropine. For labeling M_5 muscarinic cholinergic receptor, lymphocyte suspension and sections were pre-incubated in a phosphate-$MgCl_2$ buffer containing 30 μg/ml of mamba venom for 30 min at 20°C, and then incubated with 1 μM AQ-RA 741 and 0.5 nM [^{3}H]NMS for 30 min at 20°C.

At the end of incubations, cells were isolated onto Whatman GF-B glass fiber filters and processed as detailed above. The same procedure was followed for brain sections which were then processed for autoradiography.

σ_1 Receptor [10]

The binding properties of the test compounds to guinea pig σ_1 receptor were studied in guinea pig brain membranes using [^{3}H]-(+)-pentazocine as the radioligand. To each total binding assay tube were added 900 μL of the tissue suspension, 50 μL of 4.0 nM [^{3}H]-(+)-pentazocine, 50 μL Tris-HCl buffer, pH 8.0. To nonspecific binding each assay tube were added 900 μL of the tissue suspension, 50 μL of [^{3}H]-(+)-pentazocine, 50 μL of 10 μM haloperidol. To each specific binding assay tube were added 900 μL of the tissue suspension, 50 μL of [^{3}H]-(+)-pentazocine, 50 μL of compound **11b** solution in various concentrations (10^{-5} mol to 10^{-10} mol). The tubes were incubated at 25 °C for 180 min. The incubation was followed by a rapid vacuum filtration through Whatman GF/B glass filters, and the filtrates were washed twice with 5 mL of cold buffer and transferred to scintillation vials. Scintillation fluid (1.0 mL) was added, and the radioactivity bound was measured using a PE Microbeta 2450 liquid scintillation counter.

σ_2 Receptor [10]

The binding properties of the test compounds to guinea pig σ_2 receptor were studied in guinea pig brain membranes using [^{3}H]-DTG. The membranes were incubated with 3 nM [^{3}H]-DTG in the presence of 400 nM (+)-SKF-10047 to block σ1 sites. To each total binding assay tube were added 850 μL of the tissue suspension, 50 μL of 3.0 nM [^{3}H]-DTG, 50 μL of 400 nM (+)-SKF-10047, 50 μL Tris-HCl buffer, pH 8.0. To each nonspecific binding assay tube were added 850 μL of the tissue suspension, 50 μL of [^{3}H]-DTG, 50 μL of 400 nM (+)-SKF-10047, 50 μL of 10 μM DTG. To each specific binding assay tube were added 850 μL of the tissue suspension, 50

μL of [^{3}H]-DTG, 50 μL of 400 nM (+)-SKF-10047, 50 μL of compound **11b** solution in various concentrations (10^{-5} mol to 10^{-10} mol). The tubes were incubated at 25 °C for 120 min. The incubation was followed by a rapid vacuum filtration through Whatman GF/B glass filters, and the filtrates were washed twice with 5 mL of cold buffer and transferred to scintillation vials. Scintillation fluid (1.0 mL) was added, and the radioactivity bound was measured using a PE Microbeta 2450 liquid scintillation counter.

NMDA receptor [11]

Rat hippocampal membrane was homogenized in 20 volumes of ice-cold 50 mM phosphate buffer (pH 7.4) using an ULTRA TURAX homogenizer, and centrifuged twice for 10 min at 50,000g with resuspension of the pellet in fresh buffer. The final pellet was resuspended in phosphate buffer. For total binding, to each assay tube was added 900 μL of membranes, 50 μL of 1 nM [^{3}H]-glycine with 10 μM glutamic acid, and 50 μL of phosphate buffer. For nonspecific binding, to each assay tube was added 900 μL of membranes, 50 μL of 1 nM [3H]-glycine with 10 μM glutamic acid, and 50 μL of 1 μM glycine. For specific binding, to each assay tube was added 900 μL of membranes, 50 μL of 1 nM [^{3}H]-glycine with 10 μM glutamic acid, 50 μL of compound **11b** solution in various concentrations (10-5 mol to 10-10 mol). The tubes were incubated at 30 °C for 30 min. The incubation was followed by rapid vacuum filtration through Whatman GF/B glass filters, and the filtrates were washed twice with 5 mL of cold buffer and transferred to scintillation vials. 1.0 mL Scintillation fluid was added, and the radioactivity bound was measured using a PE Microbeta 2450 liquid scintillation counter.

Table 1. Binding affinities for the additional receptors of compound **11b**

Receptor	Ki (nM)
DAT	> 1000
D_1	> 1000
D_2	> 1000
D_3	> 1000
5-HT_{2A}	320
5-HT_{2C}	> 1000
α_2	> 1000
H_2	> 1000
H_4	> 1000
M_1	> 1000
M_2	> 1000
M_3	> 1000
M_4	> 1000
M_5	> 1000
σ1	> 1000
σ2	> 1000
NDMA	> 1000

4. hERG Affinity [1]

The hERG potassium channel blocking potential was evaluated using the whole-cell patch clamp method with hERG channels expressed in HEK 293 cells as the biological material. The Axopatch 200B amplifier and Digidata 1440A converter from Molecular Devices were integral to the setup. Electrodes crafted from borosilicate glass with filament by Sutter Instrument Company were used for recordings. Command pulse protocols for voltage clamping and data acquisition were managed by pCLAMP software, version 10.1. The bath solution contained 137 mM NaCl, 5.4 mM KCl, 10 mM glucose, 10 mM HEPES, and 2 mM $CaCl_2$, with pH adjusted to 7.5 by NaOH. The pipet solution was composed of 140 mM KCl, 1 mM $MgCl_2$, 5 mM EGTA, 10 mM HEPES, and 5 mM Na_2ATP, with pH adjusted to 7.2 using KOH. The compound **11b** was dissolved in a 50% (w/v) DMSO solution at an initial concentration of 1 mM and diluted to the test concentration with the bath solution.

To investigate how the steady-state blockage of hERG channels varies with different drug concentrations (0.3, 1, 3, and 10 μM) in HEK cells, the process was set so that the holding membrane potential would transition from -80 to +50 mV for a duration of 2 seconds, then revert to -50 mV for 3 seconds. This cycle was repeated every 30 seconds with a sampling rate of 4 kHz and data was low-pass filtered at 1 kHz. The tail currents were captured at -50 mV in both an unaltered condition and when the drug was added at concentrations identified through empirical means. Data acquisition was conducted employing Clampfit software, version 10.2, which is a component of the larger pCLAMP software suite, version 10.1. The hERG inhibition tests were conducted three times for consistency in results. Outcomes from these tests were then imported into the Statistical Package for the Social Sciences (SPSS) for comprehensive data analysis.

5. Acute Toxicity

The acute toxicity testing was performed in female rats according to the up-and-down procedure as previously described [12]. Briefly, a total of 6 F344 rats were orally dosed with doses of the compound **11b** (800, 640, 512, 640, 512and 410 mg/kg), one at a time, at a minimum of 48-hour intervals. The number of surviving animals was recorded after 24 h of drug administration, and the percent mortality in each group was calculated. The LD_{50} values were calculated by using the soft AOT425statpgm (Version: 1.0).

6. Behavioral Studies

6.1 Forced swimming test (FST) [3]

A total of 144 naive mice were randomly assigned to 9 groups consisting of vehicle control, duloxetine (8, 16 and 32 mg/kg), and compound **11b** (2.5, 5, 10, 20 and 40 mg/kg) with each group containing 16 mice. One hour after oral administration of test compounds, each mouse was forced to swim in an open cylindrical container (diameter of 10 cm, height of 25 cm, containing 15 cm of water with temperature maintained at 24 ± 1°C). The duration of immobility during the last 4 minutes of a total time of 6 minutes was recorded, and animals were judged to be immobile when they floated motionless, making only necessary movements to keep their heads above the water.

6.2 Tail suspension test (TST) [3]

A total of 160 naive mice were randomly assigned to 10 groups consisting of vehicle control, duloxetine (8, 16, 32 and 64 mg/kg), and compound **11b** (3, 6, 12, 24 and 48 mg/kg) with each

group consisting of 16 mice. After oral administration of compounds for 60 minutes, each mouse was suspended on the top of the apparatus using adhesive tape placed approximately 1 cm from the tip of the tail. The immobility duration of the last 4 minutes of a total time period of 6 minutes was recorded. Mice were considered to be immobile when hung passively without moving.

6.3 Locomotor activity measurement [3]

A total of 160 naive mice were randomly divided into 10 groups with 16 mice in each group. Animals were transferred into the testing room at least 2 hours prior to drug administration. Fifty-five minutes after oral administration of different doses of duloxetine (10, 20, 40 and 80 mg/kg) and compound **11b** (5, 10, 20, 40 and 80 mg/kg) or vehicle, mice were individually placed into the corner of each test chamber (length× width × height: 25 × 25× 30 cm) for a 5-minute acclimation period. Sixty minutes after drug administration, spontaneous locomotor activity was recorded for 15 minutes using a tracking and computerized analysis system (Clever Sys Inc., Leesburg, VA). The group of MK-801 was administered by intraperitoneal injection (0.3 mg/kg). Thirty minutes after intraperitoneal administration, spontaneous locomotor activity was recorded. The area was wiped and cleaned with a 75% alcohol solution and dried before each test.

7. Pharmacokinetics Study [1]

The HPLC conditions were as follows: Shimadzu LC30AD spectrometer； column, SHIMADZU C18 (2.1 mm×50 mm, 2 μm); mobile phase A: 0.1% FA-Acetonitrile; mobile phase B: 0.1% FA-Deionized water; flow rate, 0.4 mL/min; column temperature: 25 °C; UV detection was performed at 210 nm. The Mass conditions were as follows: ESI (positive), Ion spray voltage: 5500V, Curtain gass: 30, Temperature: 550°C, Ion Source Gas1: 55, Collision Gas: 7. For routine compound **11b** screening, rats (n = 6/group) were dosed via the lateral tail vein at the indicated dose for iv administration (2 mg/kg, NS) or via oral gavage (20 mg/kg, Deionized water). At 0, 0.08, 0.25, 0.5,1、2、4、7、10 and 24h after administration, serial blood samples were collected from the lateral tail vein into heparinized collection tubes (approximately 0.25 mL). The plasma was separated by centrifugation, and the sample was prepared for LC/MS analysis by protein precipitation with acetonitrile. The plasma samples were analyzed for drug and internal standard via the LCMS/MS protocol.

8. ^{1}H NMR of Compound 11b

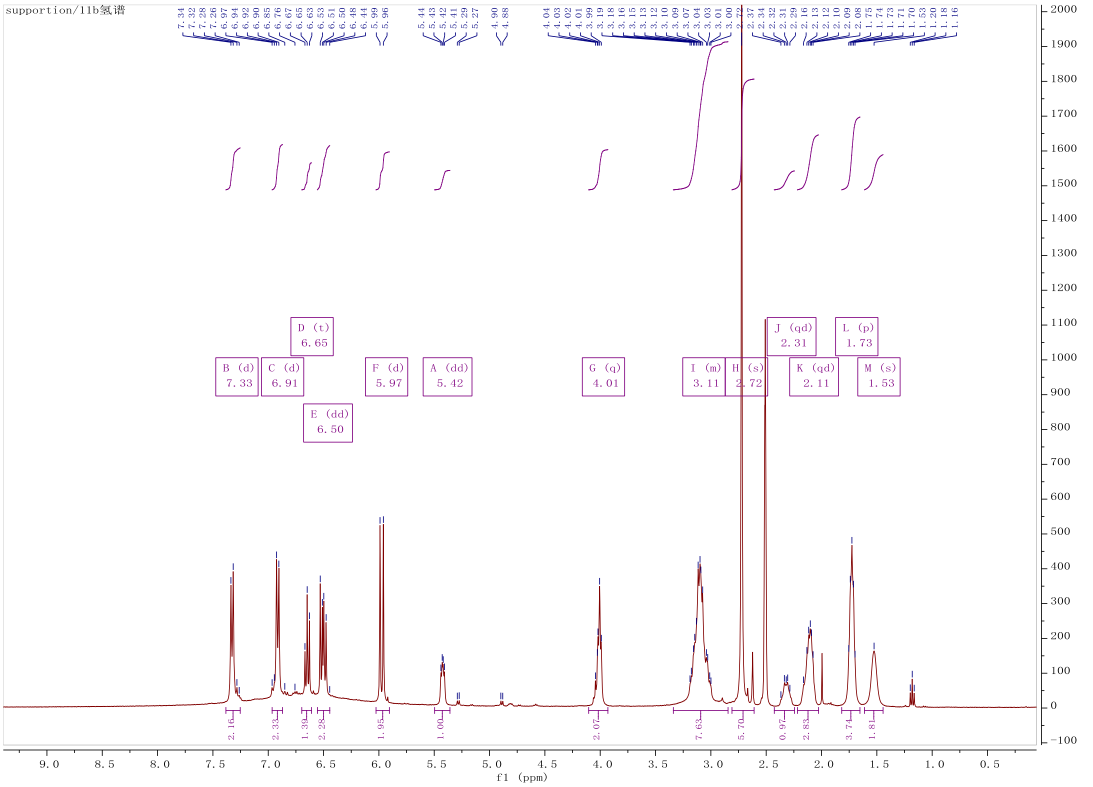

^{13}C NMR of Compound 11b

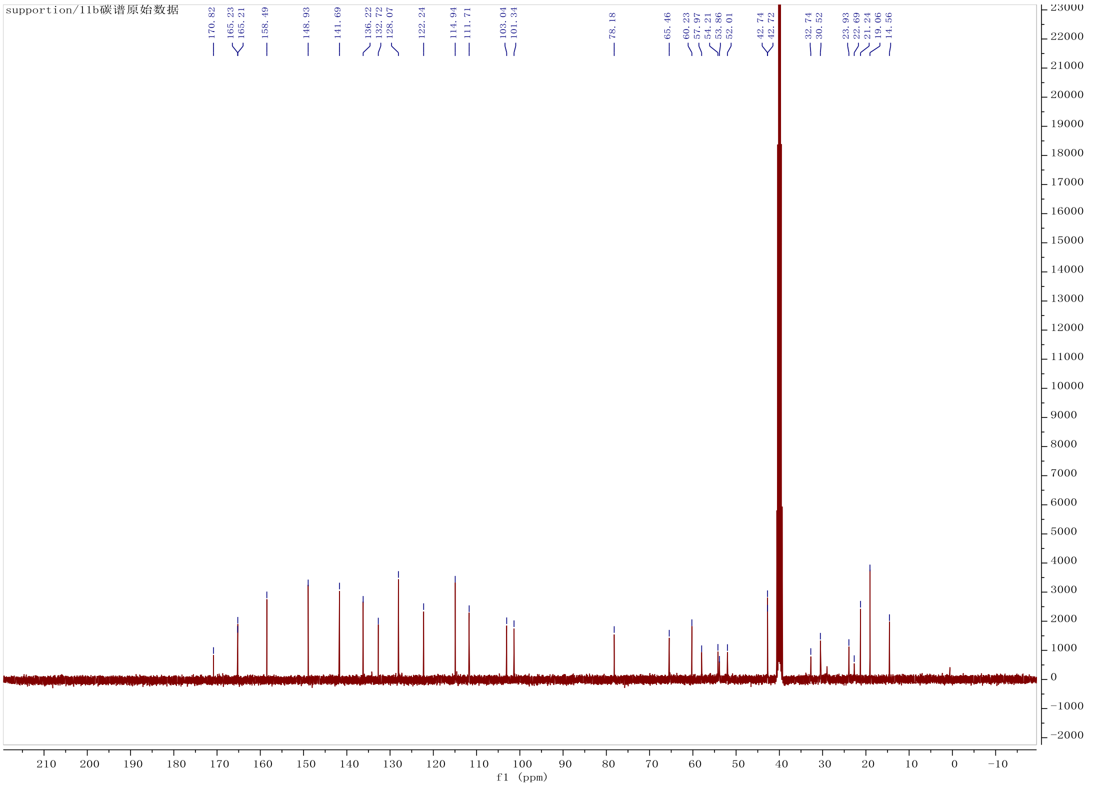

HRMS analysis of Compound 11b

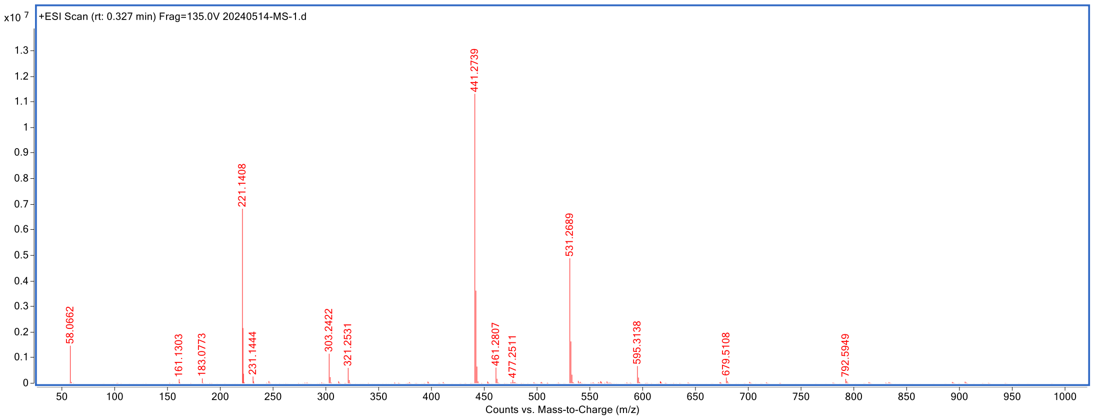

9. ^{1}H NMR of other Target Compounds

Compound 11a

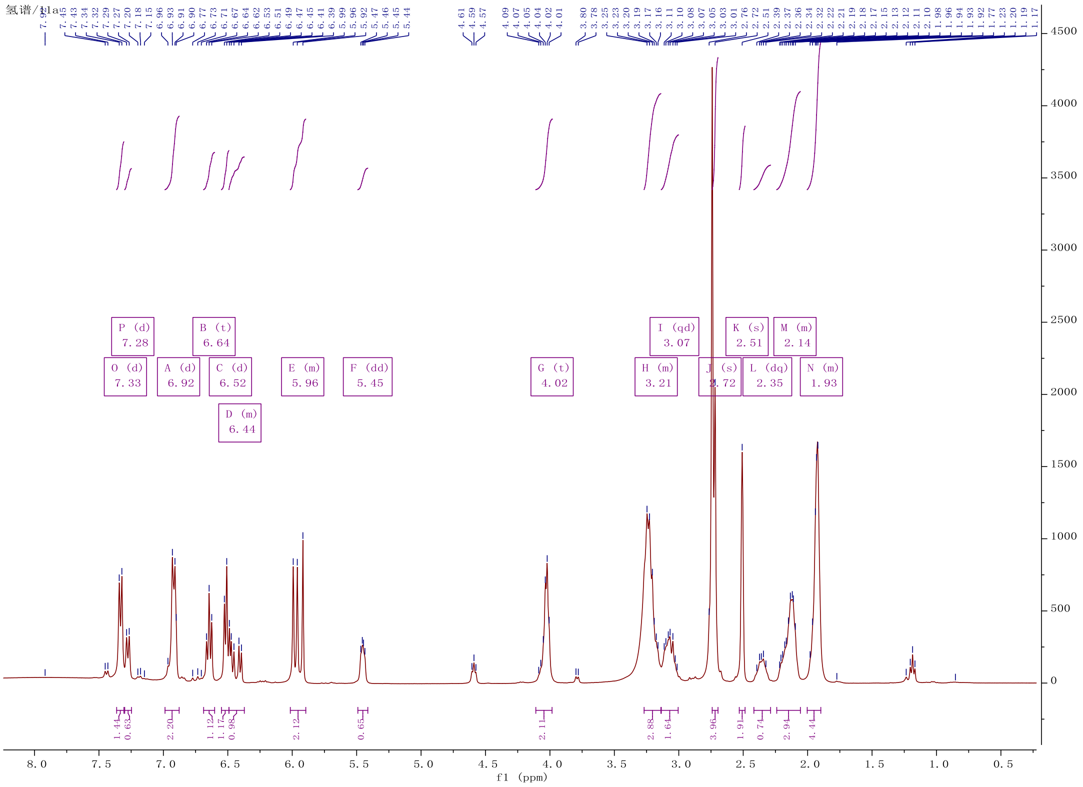

Compound 11c

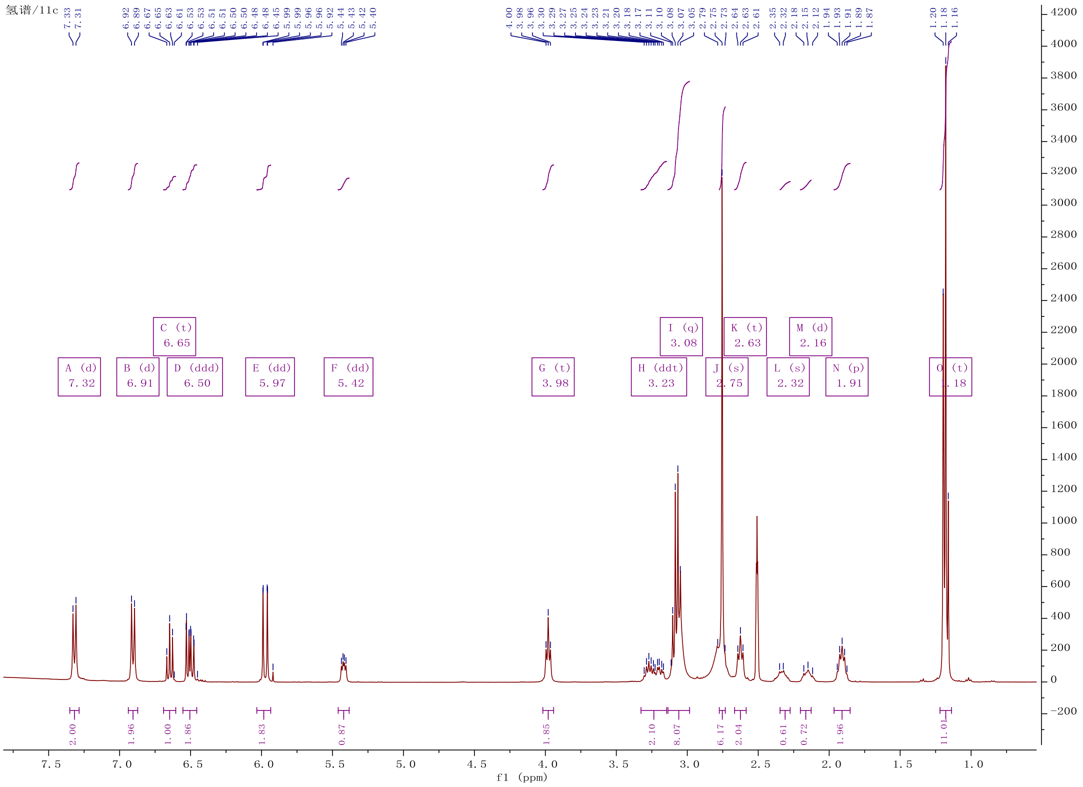

Compound 11d

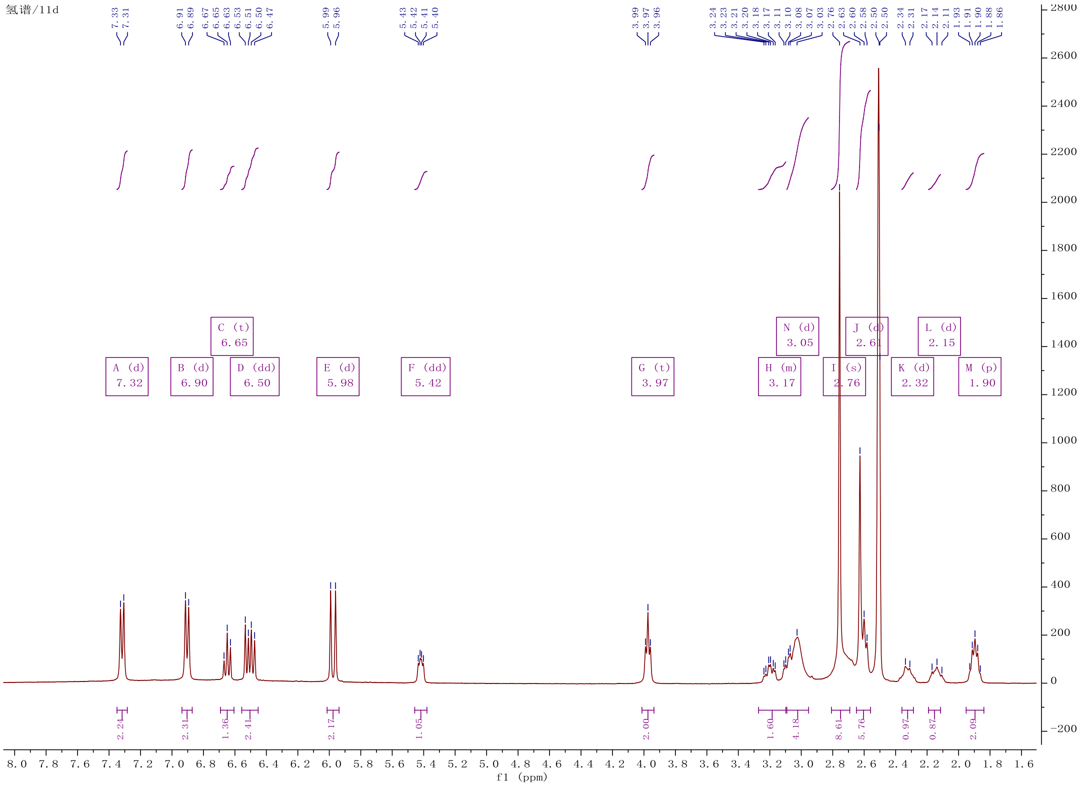

Compound 11e

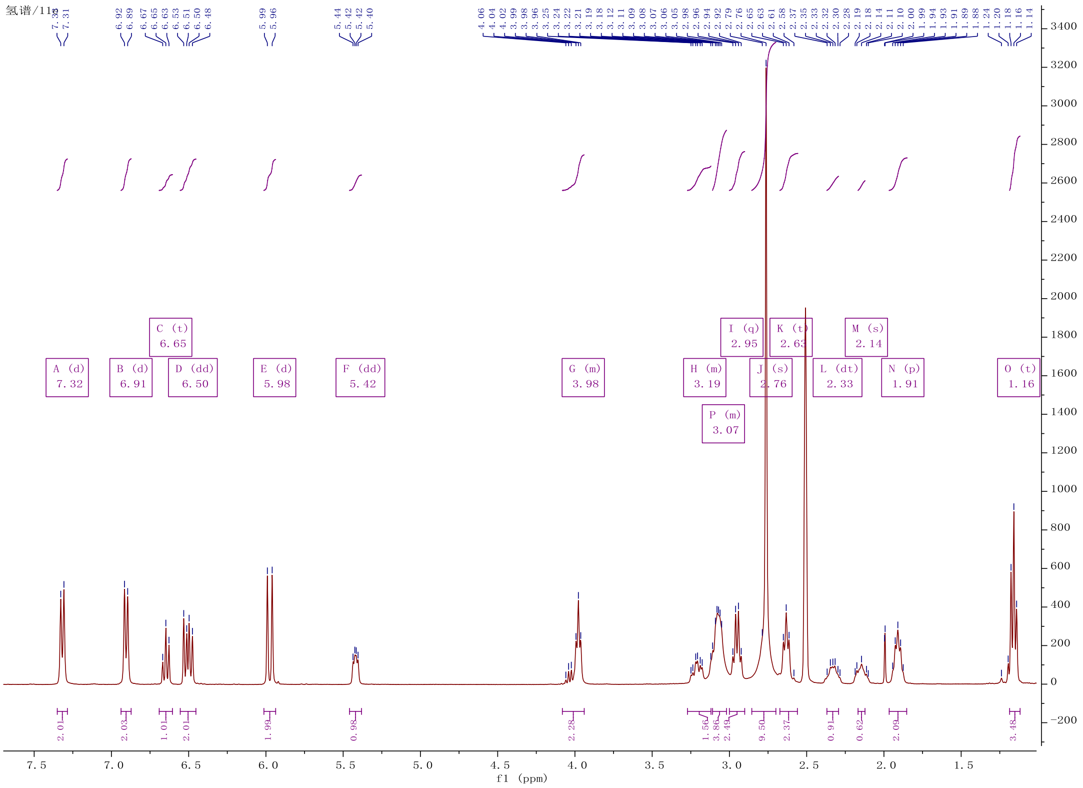

Compound 11f

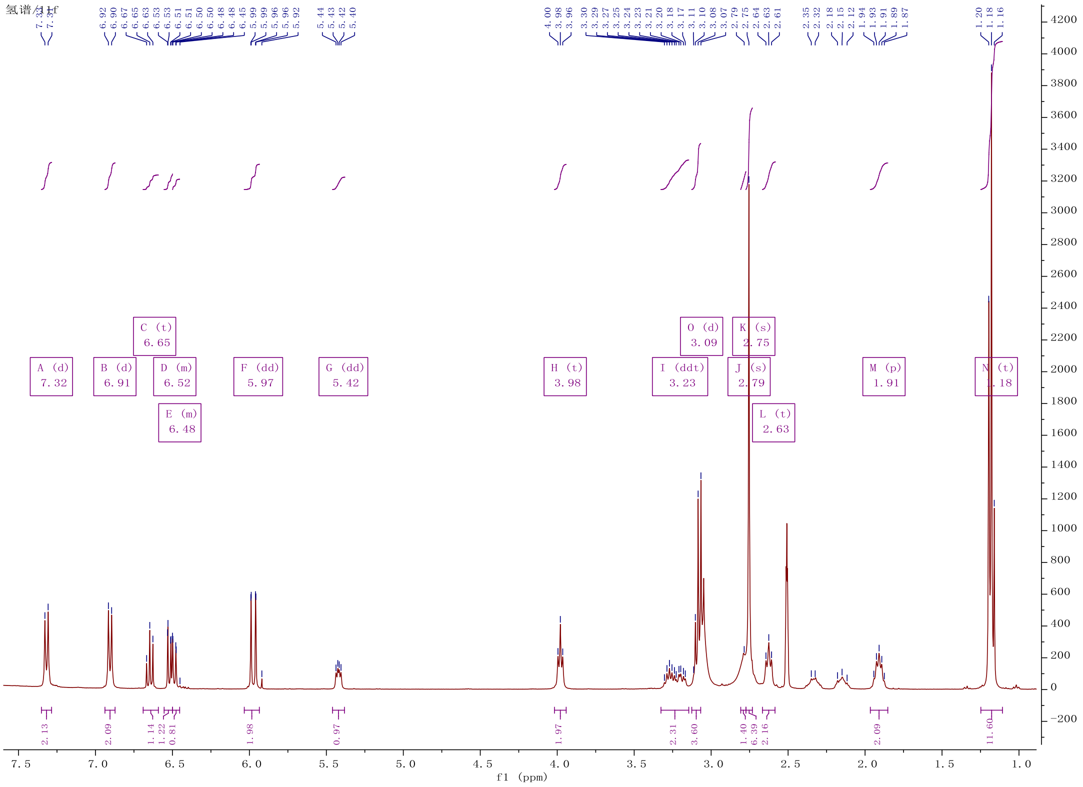

Compound 11g

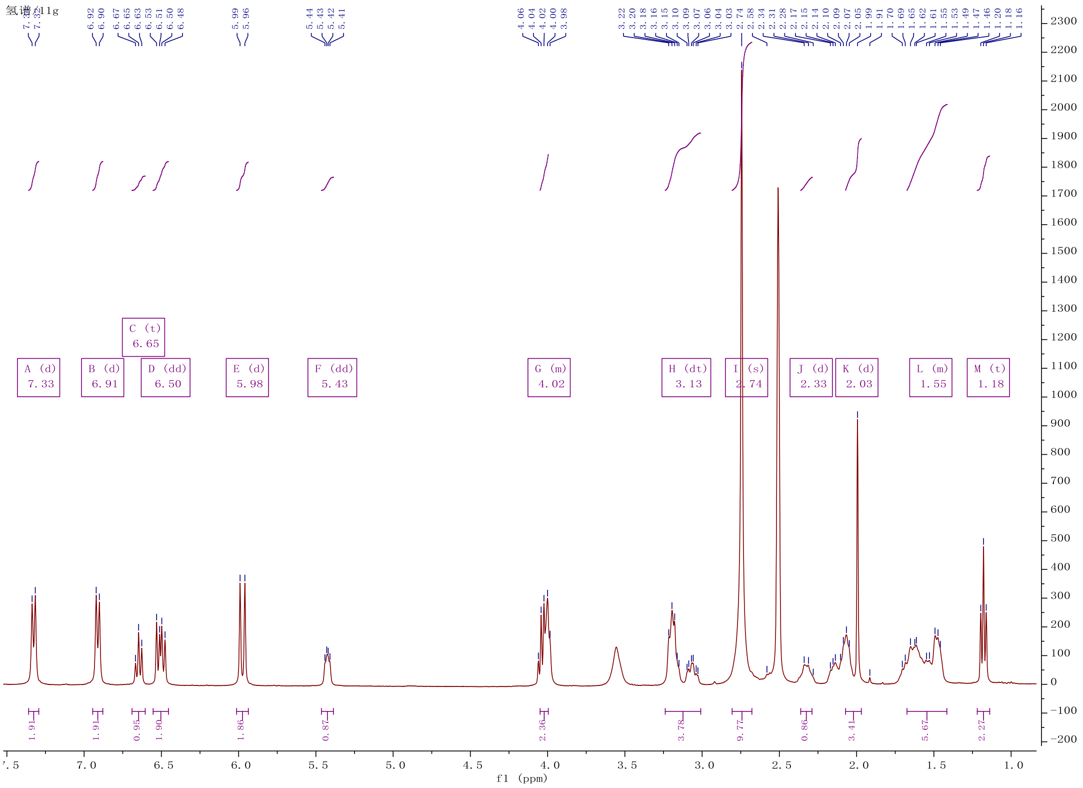

Compound 11h

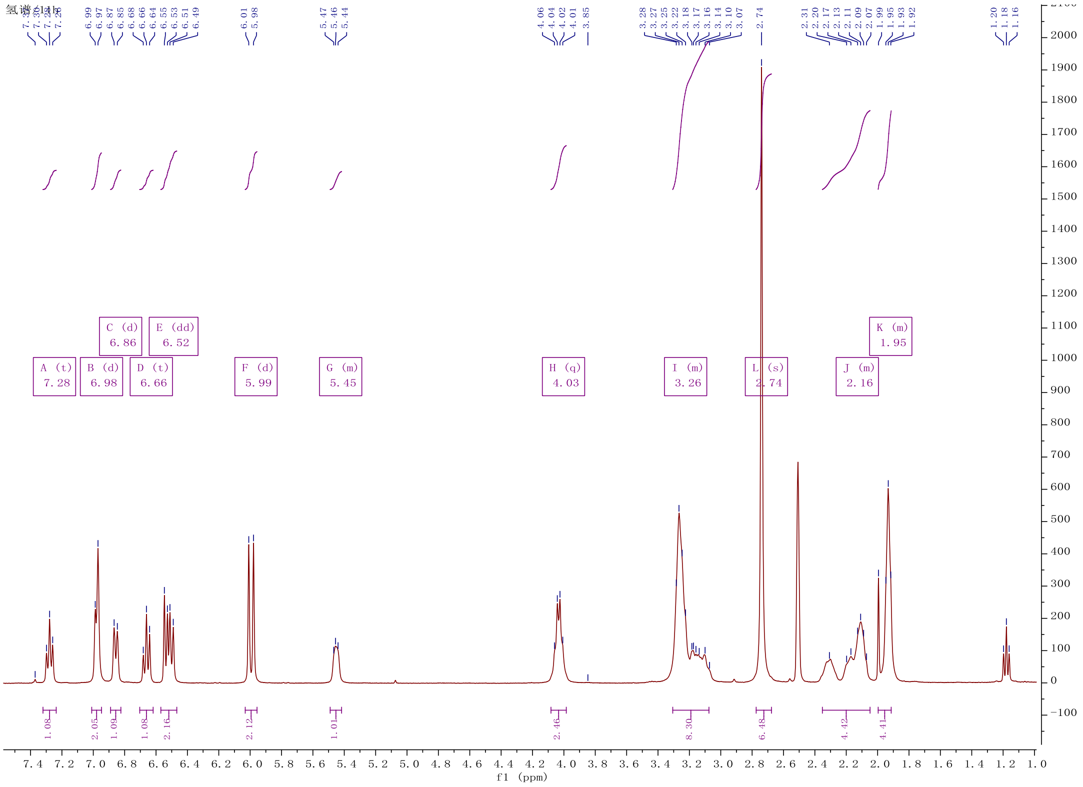

Compound 11i

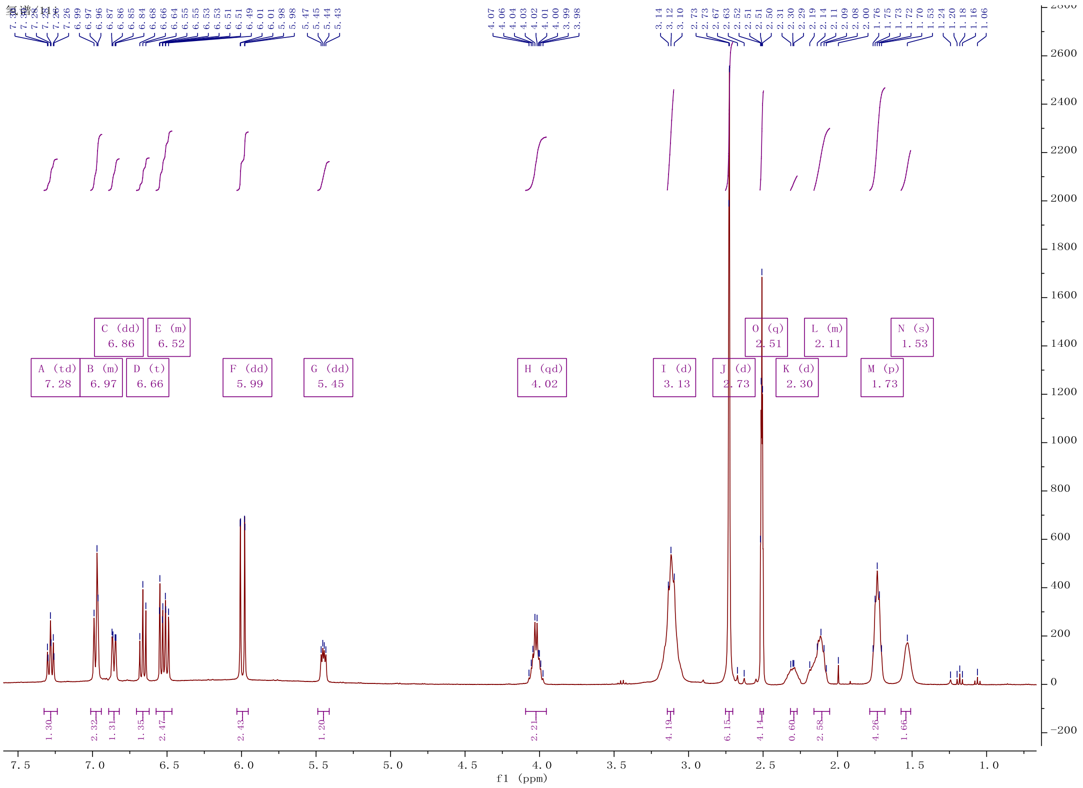

Compound 11j

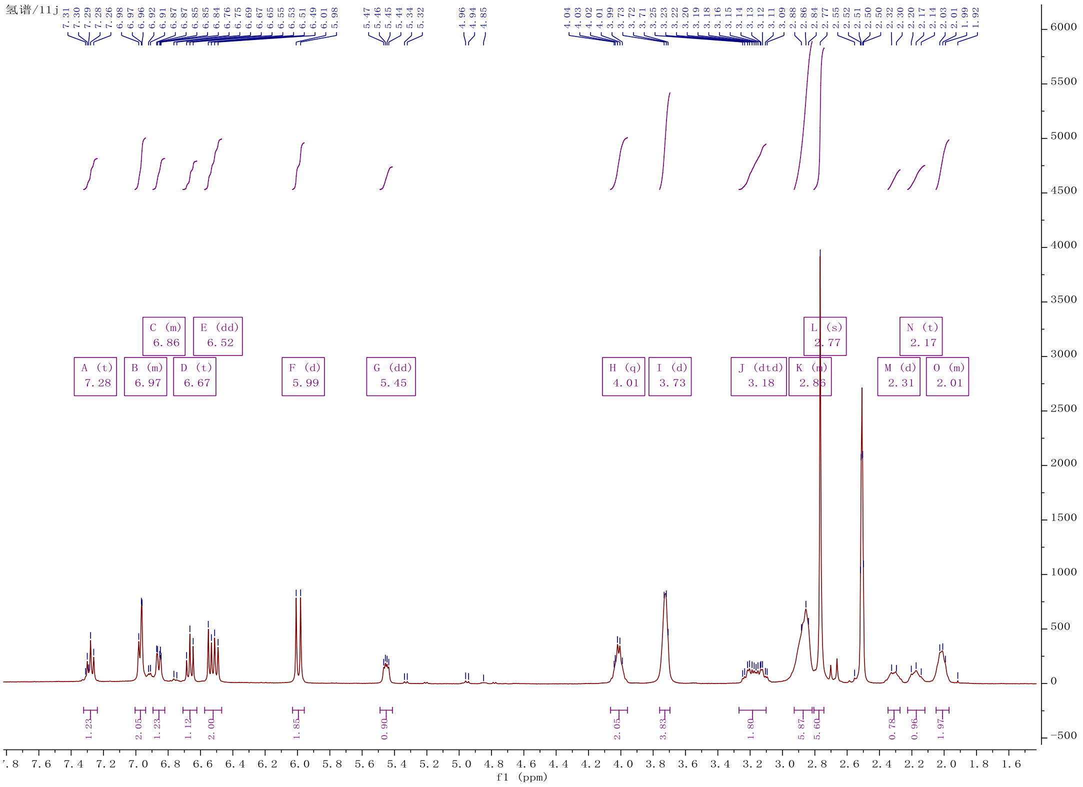

Compound 11k

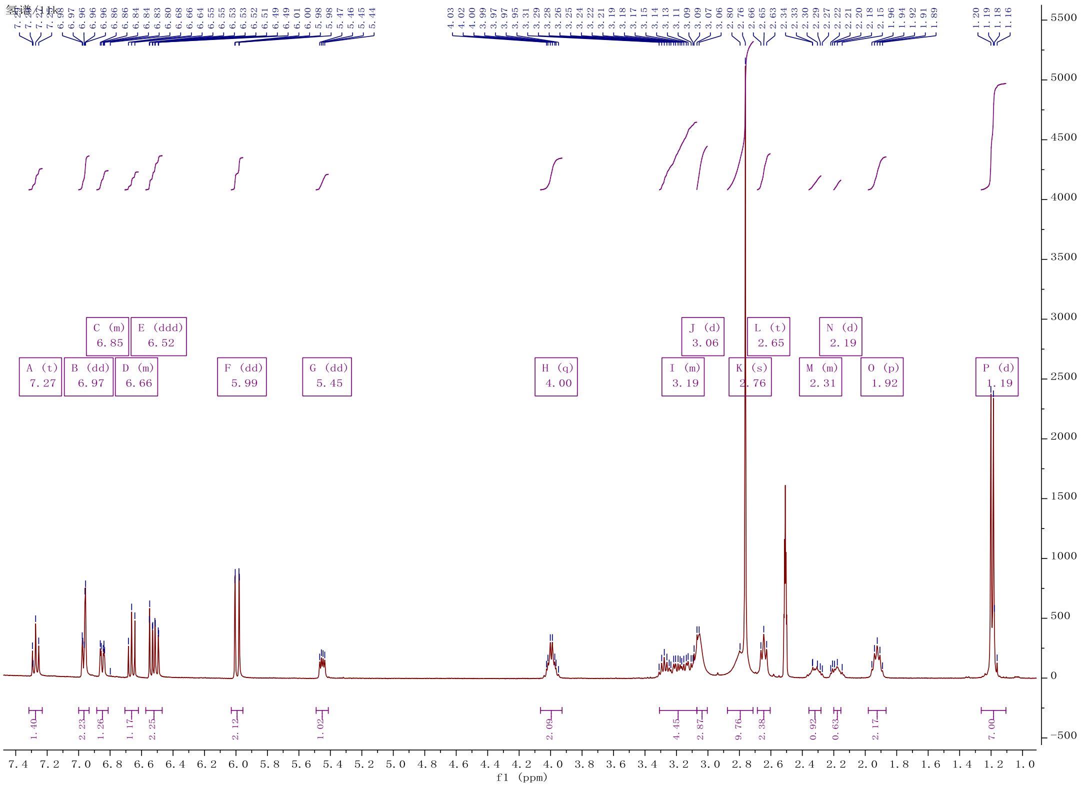

Compound 11l

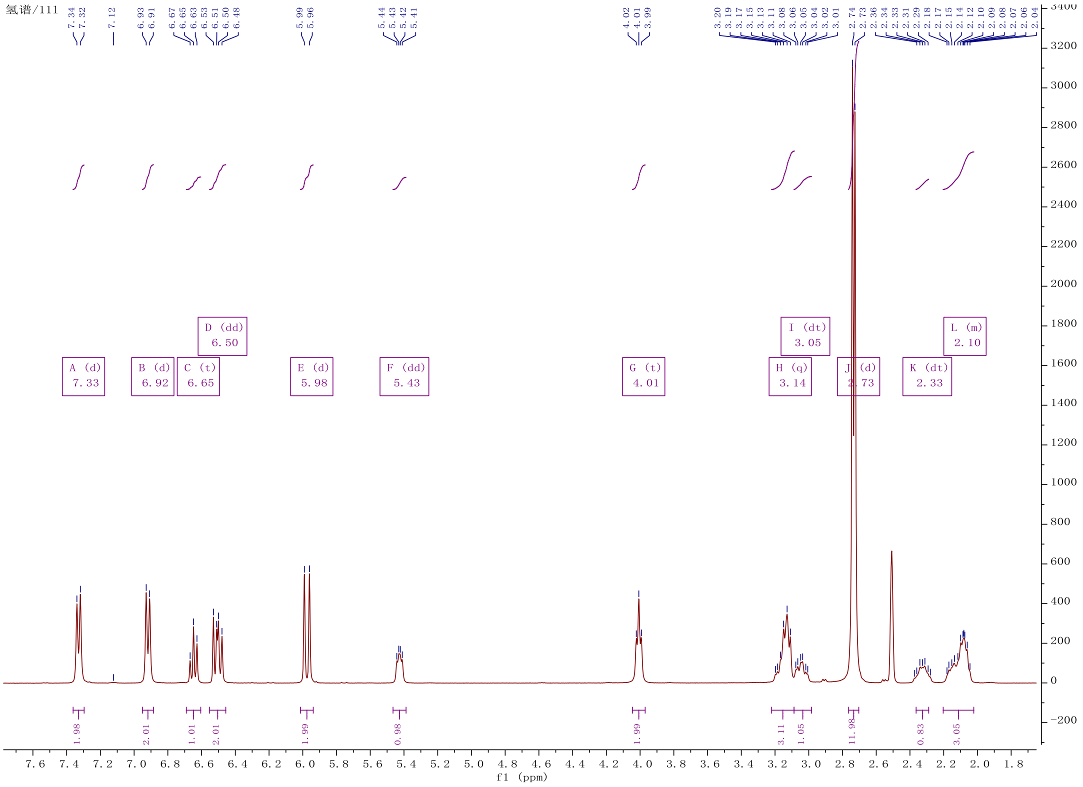

Compound 11m

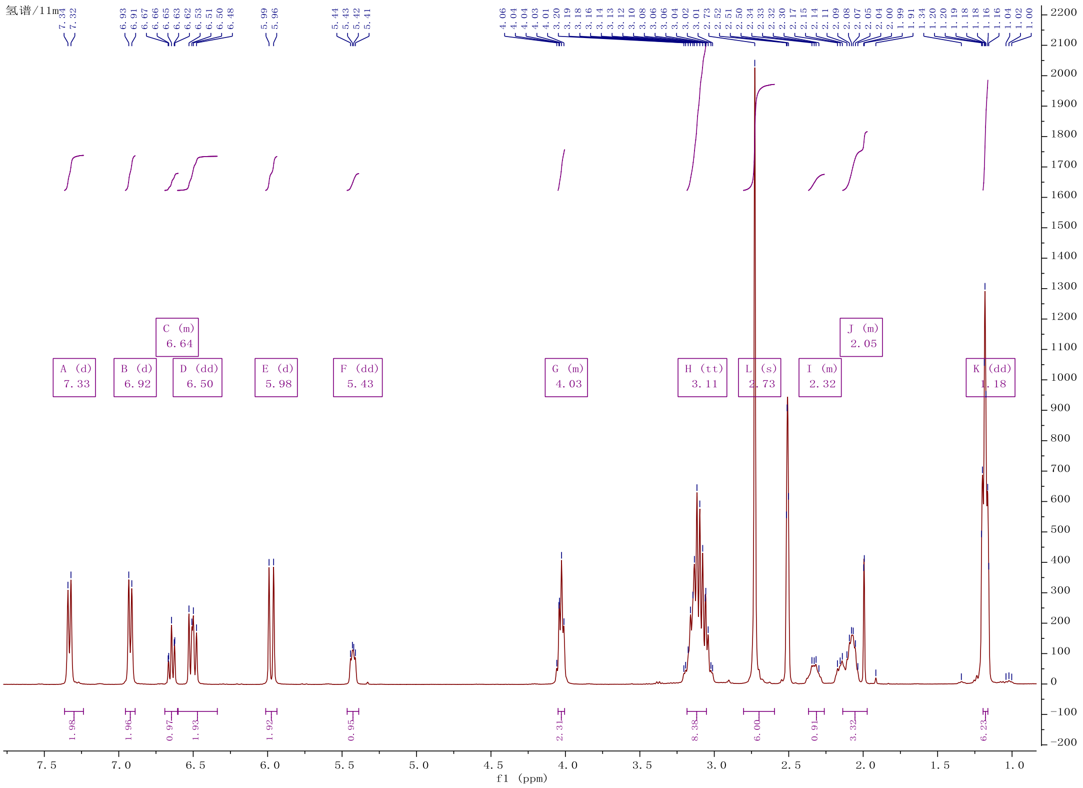

Compound 11n

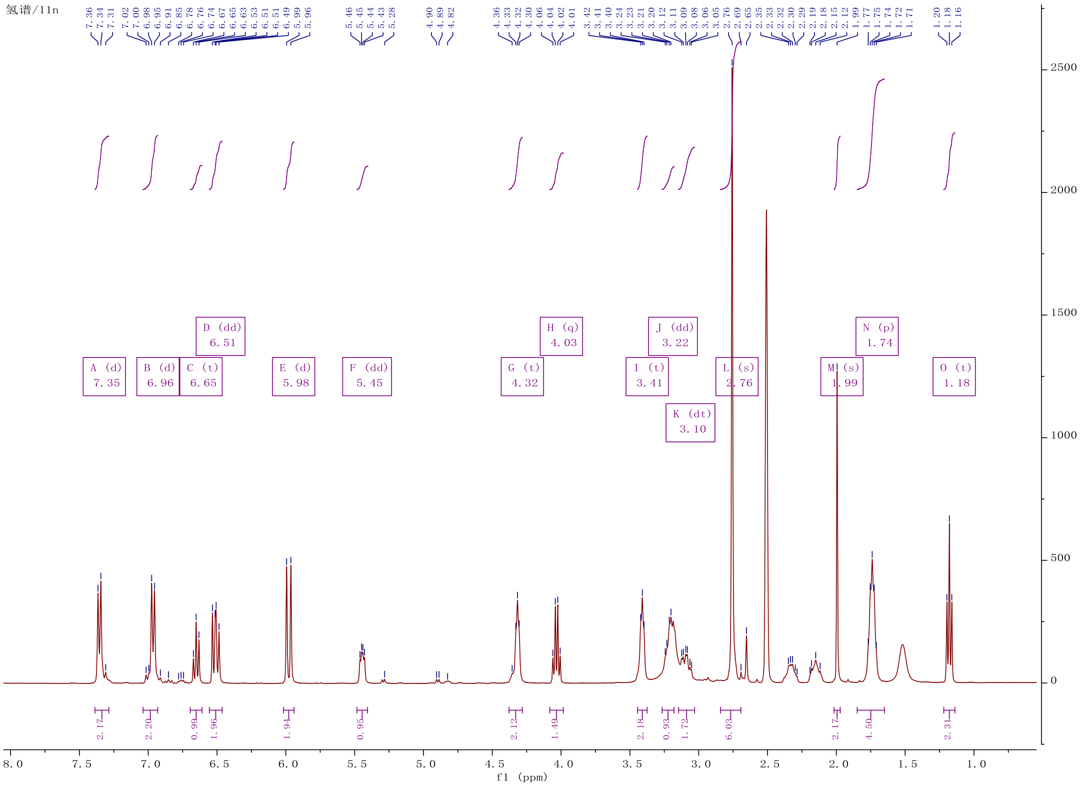

Compound 11o

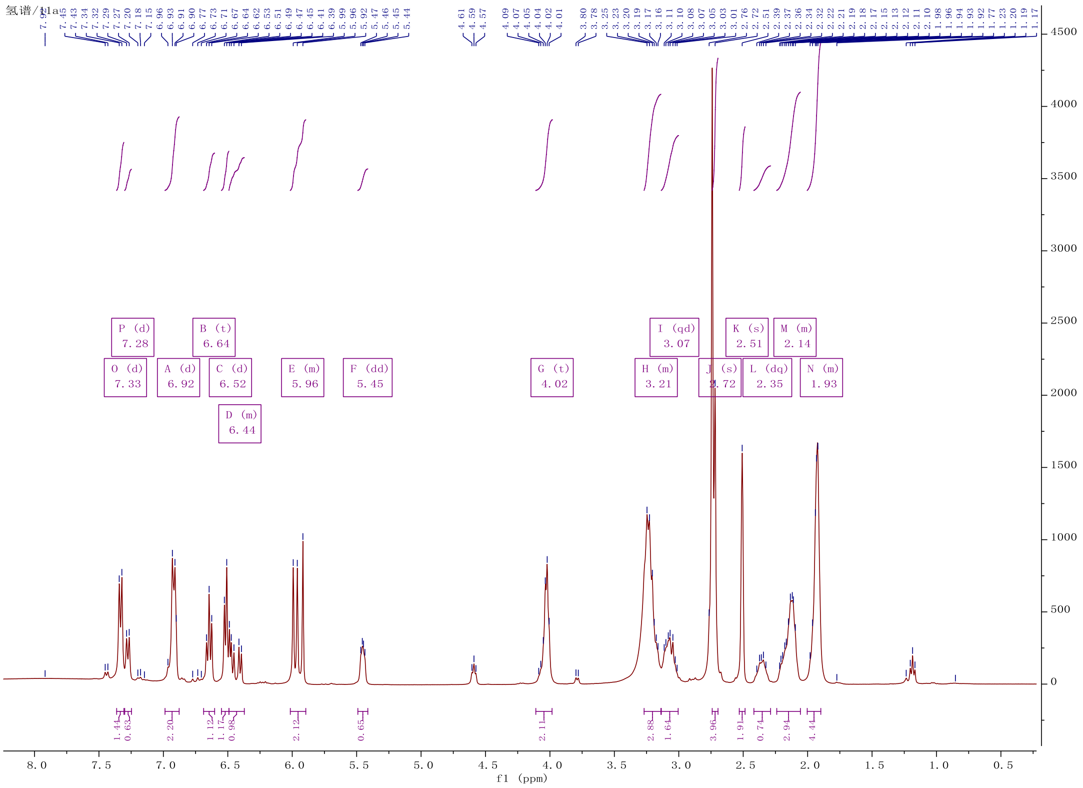

Compound 11p

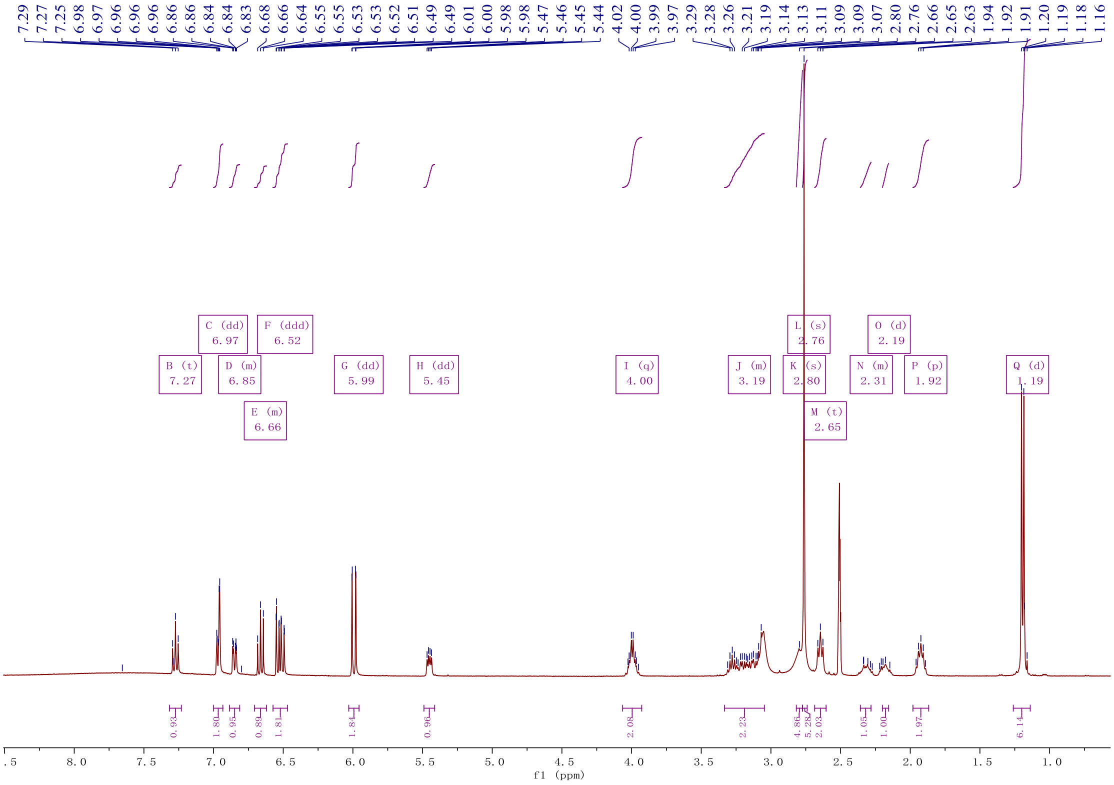

Compound 11q

氢谱/11q

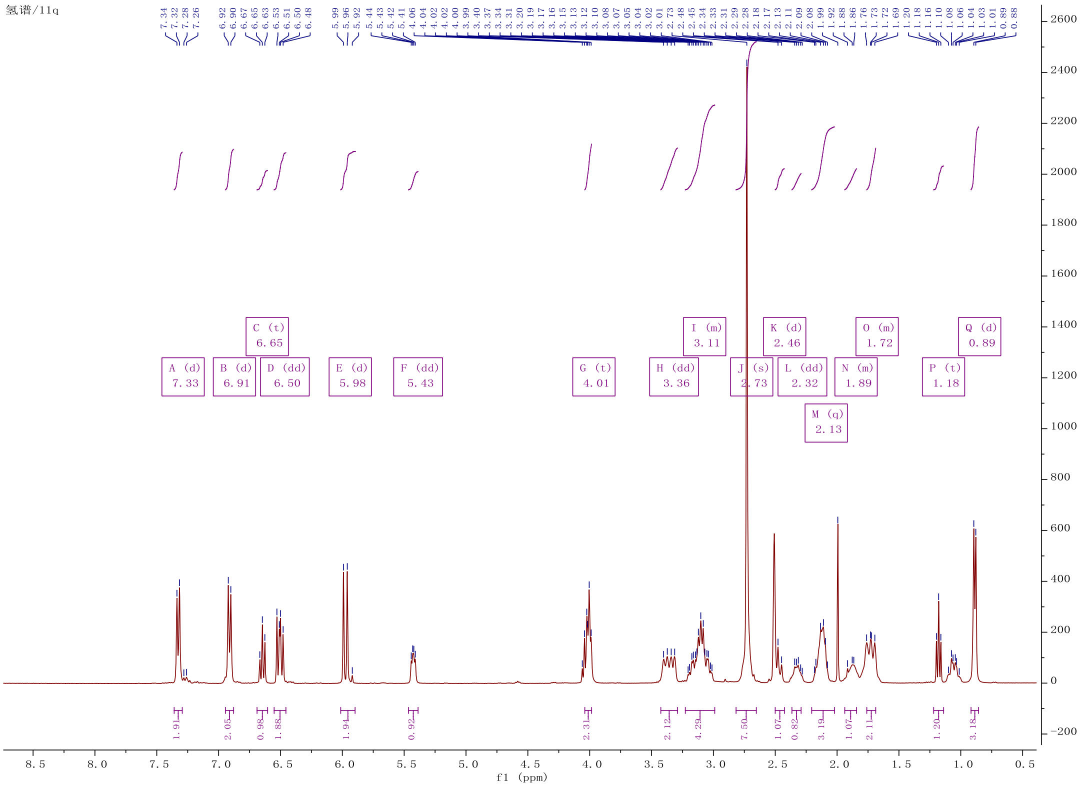

Compound 11r

氢谱/11r

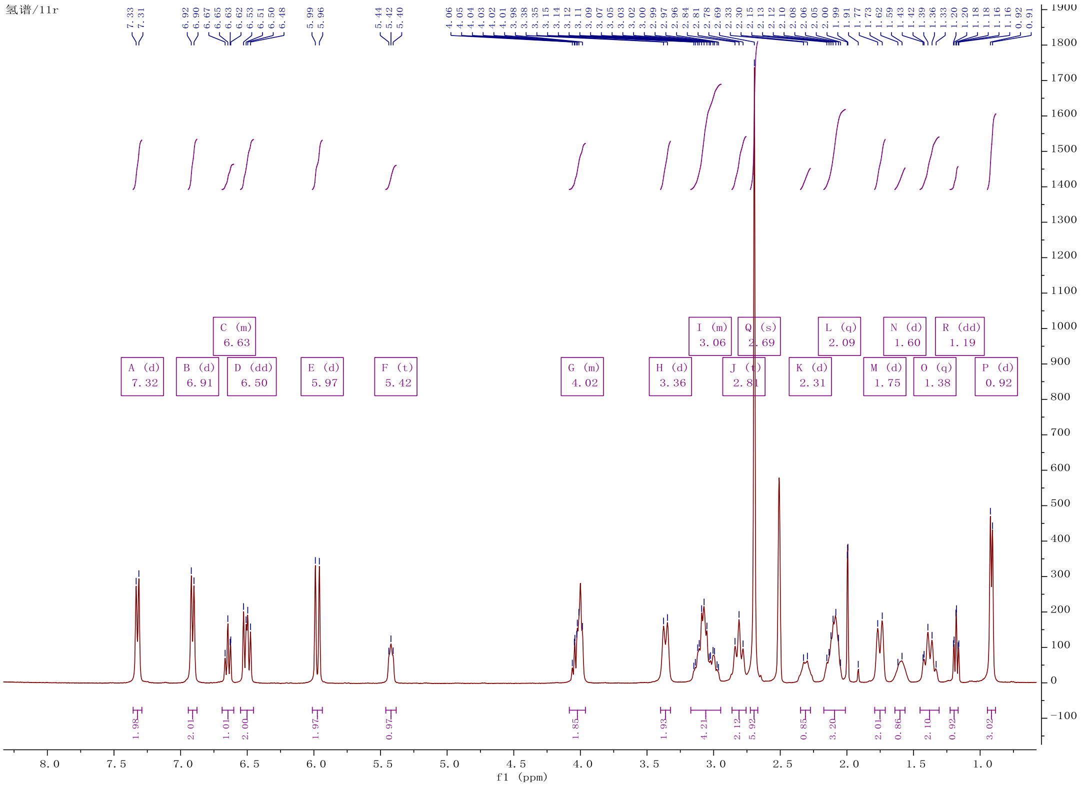

Compound 11s

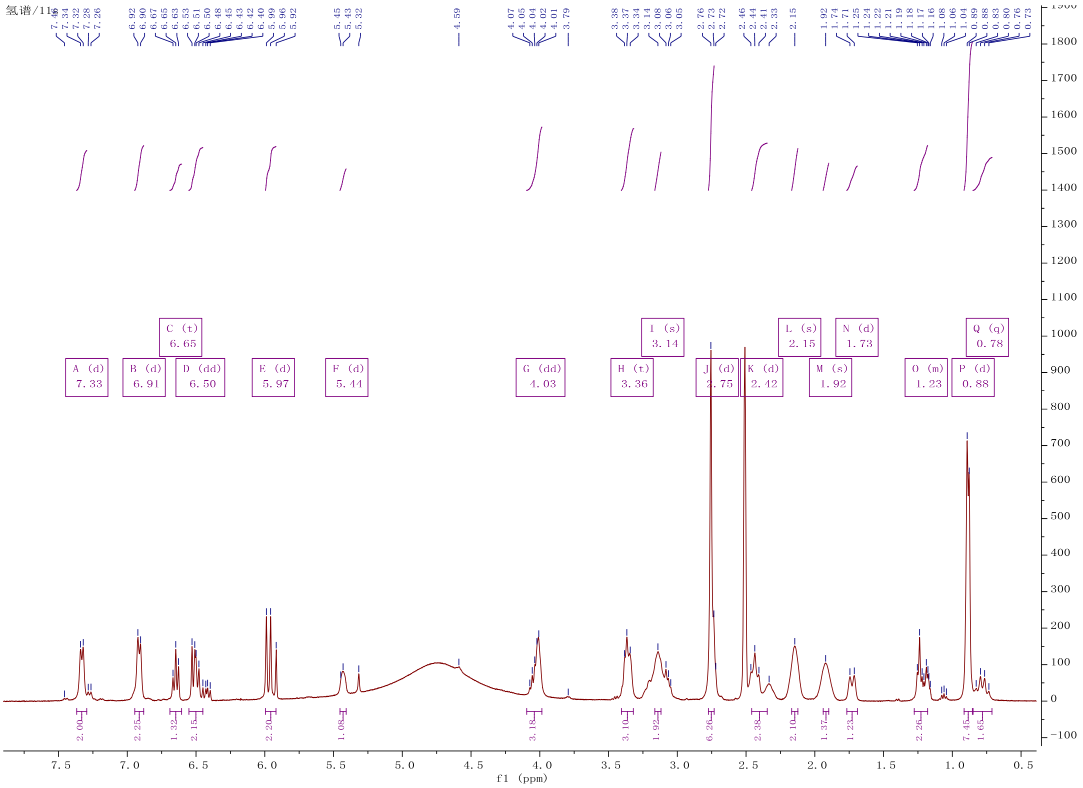

Compound 11t

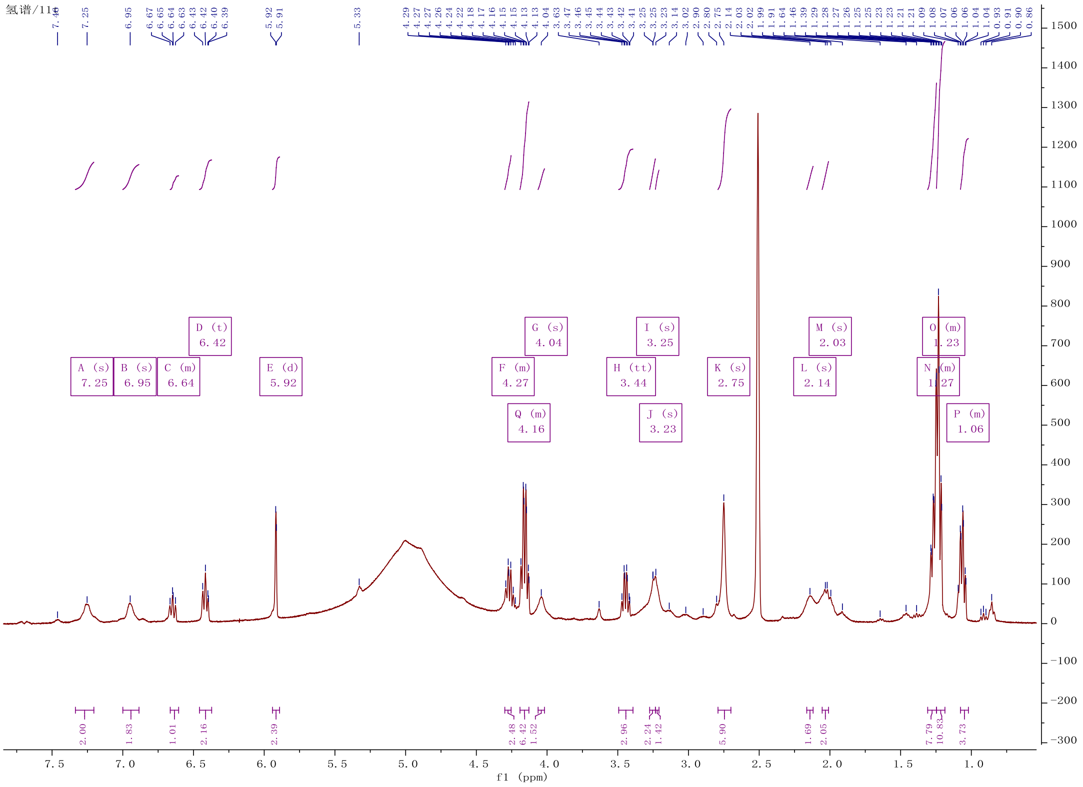

Compound 11u

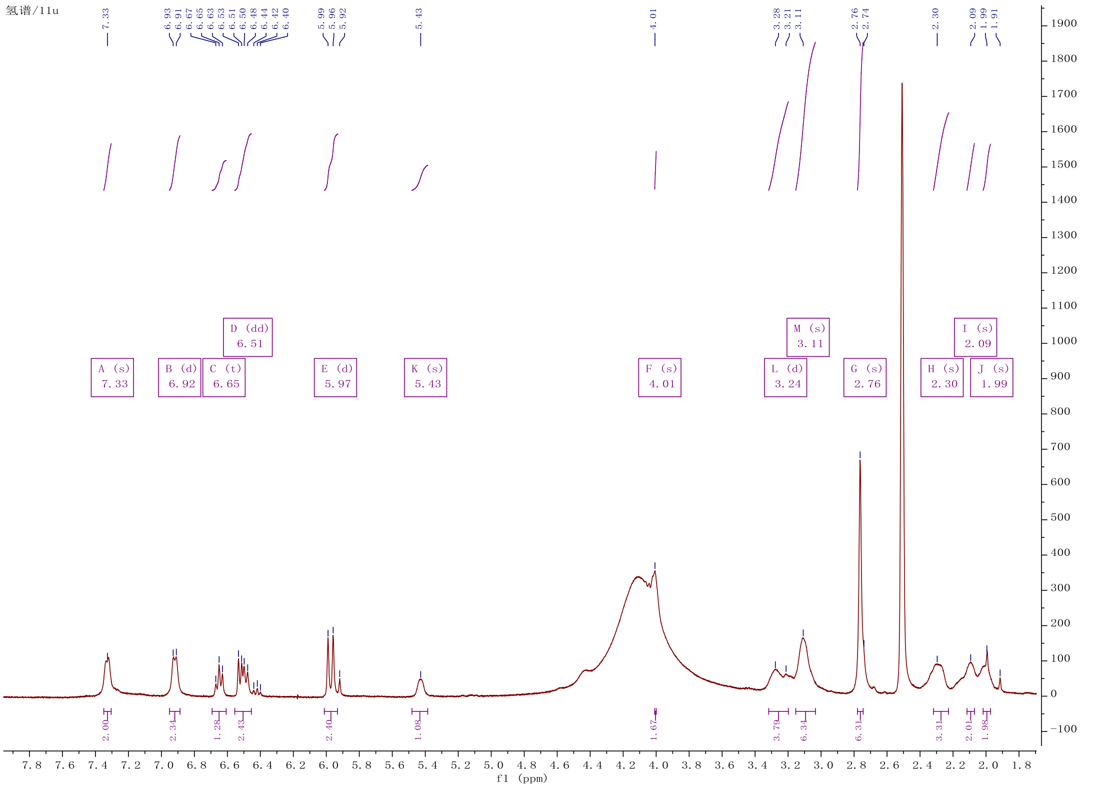

Compound 11v

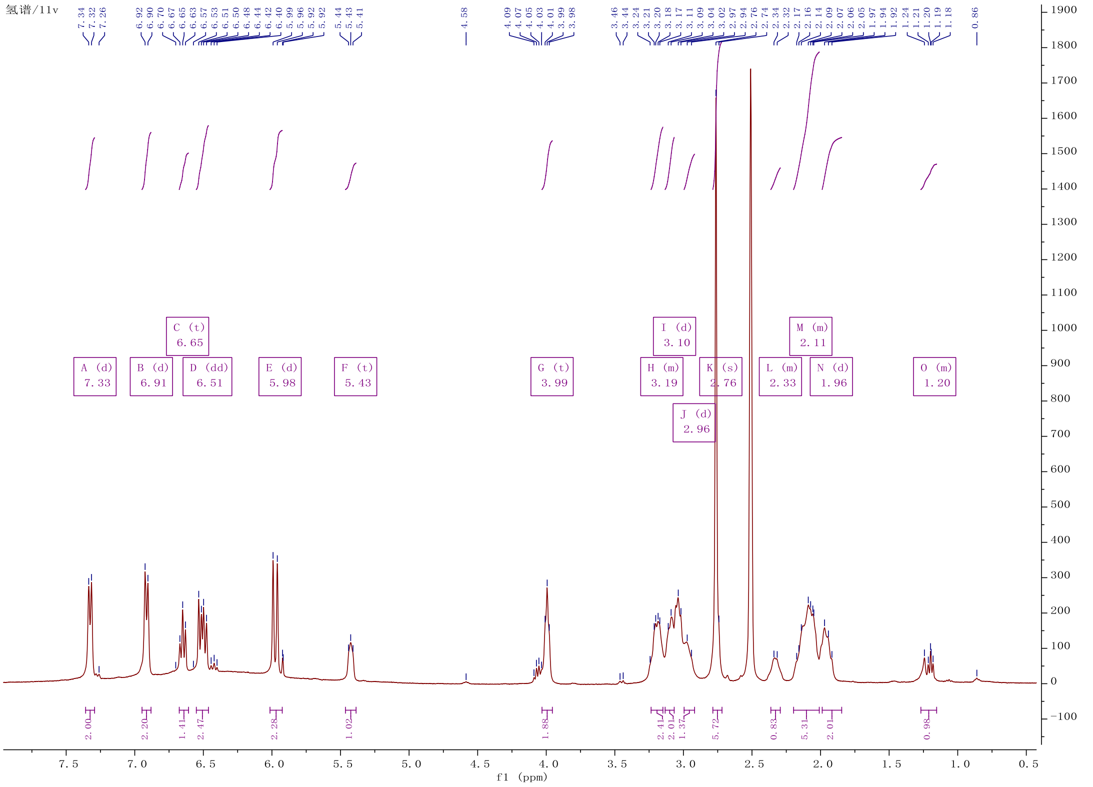

10. References

1. Jin, J.; Zhang, K.; Dou, F.; Hao, C.; Zhang, Y.; Cao, X.; Gao, L; Xiong, J.; Liu, X.; Liu, B.F.; Zhang, G.; Chen, Y. Isoquinolinone derivatives as potent CNS multi-receptor D_2/5-HT_{1A}/5-HT_{2A}/5-HT_6/5-HT_7 agents: Synthesis and pharmacological evaluation. *Eur J Med Chem*. **2020**, 207, 112709.
2. Marlon, C.; Gin, H.; Lawrence, A. B.; Zhan, C. C.; Erica, J. G.; Madhavi, P.; Marina, S.; Arlene, M.; Tracy, C.; Jill, W.; Anthony, L.; Gilbert, D.; Tiffany, G.; Jorge, D. B. Pharmacological characterization of A-960656, a histamine H_3 receptor antagonist with efficacy in animal models of osteoarthritis and neuropathic pain. *Eur. J. Pharm*. **2012**, *684*, 87-94.
3. Xu X, Wei Y.; Guo Q.; Zhao S.; Liu Z.; Xiao T.; Liu Y.; Qiu Y.; Hou Y.; Zhang G.; Wang K. Pharmacological Characterization of H05, a Novel Serotonin and Noradrenaline Reuptake Inhibitor with Moderate 5-HT_{2A} Antagonist Activity for the Treatment of Depression. *J Pharmacol Exp Ther*. **2018**, 365, 624-635.
4. Raddatz, R.; Hudkins, R. L.; Mathiasen, J. R.; Gruner, J. A.; Flood, D. G.; Aimone, L. D.; Le, S.; Schaffhauser, H.; Duzic, E.; Gasior, M.; Bozyczko-Coyne, D.; Marino, M. J.; Ator, M. A.; Bacon, E. R.; Mallamo, J. P.; Williams, M., CEP-26401 (irdabisant), a potent and selective histamine H_3 receptor antagonist/inverse agonist with cognition-enhancing and wake-promoting activities. *J Pharmacol Exp Ther*. **2012**, *340*, 124-133.
5. Fabrizio M.; Paolo C.; Roberto. A.; Roberto B.; Barbara B.; Michela B.; Letizia B.; Giorgio B.; Simone B.; Anna C.; Silvia D.; Romano D. F.; Elettra F.; Stefano F.; Carla M.; Doug M.; Michele N.; Beatrice O.; Kevin D. R.; Ilaria S.; Giovanna T.; Luca T.; Silvia T.; Filippo V.; Alessandro Z.; Laura Z. 1-(Aryl)-6-[alkoxyalkyl] -3-azabicyclo[3.1.0]hexanes and 6-(Aryl)-6-[alkoxyalkyl]-3- azabicyclo[3.1.0] hexanes: A New Series of Potent and Selective Triple Reuptake Inhibitors. *J. Med. Chem*. **2010**, *53*, 2534-2551.
6. Frecentese, F.; Fiorino, F.; Perissutti, E.; Severino, B.; Magli, E.; Esposito, A.; De Angelis, F.; Massarelli, P.; Nencini, C.; Viti, B.; Santagada, V.; Caliendo, G. Efficient microwave combinatorial synthesis of novel indolic arylpiperazine derivatives as serotoninergic ligands. *Eur. J. Med. Chem.* **2010,** 45, 752-759.
7. Cao, X.; Zhang, Y.; Chen, Y.; Oiu, Y.; Yu, M.; Xu, X,; Liu, X.; Liu, B. F.; Zhang, G. Synthesis and biological evaluation of fused tricyclic heterocycle piperazine (piperidine) derivatives as potential Multireceptor atypical Antipsychotics. *J. Med. Chem*. **2018**, 61, 10017-10039.
8. Kagermeier, N.; Werner, K.; Keller, M.; Baumeister, P.; Bernhardt, G.; Seifert, R.; Buschauer A. Dimeric carbamoylguanidine-type histamine H_2 receptor ligands: A new class of potent and selective agonists. *Bioorg Med Chem*. **2015**, 23, 3957-69.
9. Tayebatim S.K.; Codini, M.; Gallai, V.; Mannino, F.; Parnetti, L.; Ricci, A.; Sarchielli, P.; Amenta, F. Radioligand binding assay of M_1-M_5 muscarinic cholinergic receptor subtypes in human peripheral blood lymphocytes. *J Neuroimmunol*. **1999**,99, 224-229.
10. Cao, X.; Chen, Y.; Zhang, Y.; Qiu, Y.; Yu, M.; Xu, X.; Liu, X.; Liu, B. F.; Zhang, G. Synthesis and biological evaluation of new 6-hydroxypyridazinone benzisoxazoles: Potential multi-receptor-targeting atypical antipsychotics. *Eur. J. Med. Chem*. **2016**, 124, 713-728.
11. Hans-Peter, B.; Carsten, D. S., Ralf, Steinmetz, Ina, F.; Michael, L. B.; Rudolf, G.; Joachim, L.; Michael, K.; Dieter, S.; Christian, R. N. Synthesis of Thieno[2,3-b]Pyridinones Acting as Cytoprotectants and as Inhibitors of [^{3}H]Glycine Binding to the N-Methyl-D-aspartate (NMDA) Receptor. *J. Med. Chem*. **2006**, *49*, 864-871.
12. Bruce, R. D. An up-and-down procedure for acute toxicity testing, *Fundam Appl Toxicol*. **1985**, 5, 151-157.
